# DUX4 expression activates JNK and p38 MAP kinases in myoblasts

**DOI:** 10.1242/dmm.049516

**Published:** 2022-10-31

**Authors:** Christopher M. Brennan, Abby S. Hill, Michael St. Andre, Xianfeng Li, Vijaya Madeti, Susanne Breitkopf, Seth Garren, Liang Xue, Tamara Gilbert, Angela Hadjipanayis, Mara Monetti, Charles P. Emerson, Robert Moccia, Jane Owens, Nicolas Christoforou

**Affiliations:** ^1^ Rare Disease Research Unit, Pfizer Inc., Cambridge, MA 02139, USA; ^2^ WRDM Postdoctoral Program, Pfizer Inc., Cambridge, MA 02139, USA; ^3^ NGS Technology Center, Inflammation and Immunology Research Unit, Pfizer, Cambridge, MA 02139, USA; ^4^ Proteomics Technology Center, Internal Medicine Research Unit, Pfizer, Cambridge, MA 02139, USA; ^5^ Machine Learning and Computational Science, Pfizer Inc., Cambridge, MA 02139, USA; ^6^ High Content Imaging Technology Center, Internal Medicine Research Unit, Pfizer, Cambridge, MA 02139, USA; ^7^ Wellstone Muscular Dystrophy Program, Department of Neurology, University of Massachusetts Medical School, Worcester, MA 01655, USA

**Keywords:** MAP kinase signaling, Muscular dystrophy, Phosphoproteomics

## Abstract

Facioscapulohumeral muscular dystrophy (FSHD) is caused by misexpression of the DUX4 transcription factor in skeletal muscle that results in transcriptional alterations, abnormal phenotypes and cell death. To gain insight into the kinetics of DUX4-induced stresses, we activated DUX4 expression in myoblasts and performed longitudinal RNA sequencing paired with proteomics and phosphoproteomics. This analysis revealed changes in cellular physiology upon DUX4 activation, including DNA damage and altered mRNA splicing. Phosphoproteomic analysis uncovered rapid widespread changes in protein phosphorylation following DUX4 induction, indicating that alterations in kinase signaling might play a role in DUX4-mediated stress and cell death. Indeed, we demonstrate that two stress-responsive MAP kinase pathways, JNK and p38, are activated in response to DUX4 expression. Inhibition of each of these pathways ameliorated DUX4-mediated cell death in myoblasts. These findings uncover that the JNK pathway is involved in DUX4-mediated cell death and provide additional insights into the role of the p38 pathway, a clinical target for the treatment of FSHD.

## Teaser

**Summary:** A multi-omics approach shows that MAP kinase pathways are activated in response to DUX4 expression and contribute to DUX4-mediated cell death.

## INTRODUCTION

DUX4 is a double homeobox transcription factor encoded within D4Z4 macrosatellite repeat regions, and is normally expressed during embryonic development as well as in the testis and thymus of adult humans (Das and Chadwick, 2016; Hendrickson et al., 2017; Snider et al., 2010). Its expression during human development is restricted to the four-cell stage, during which it coordinates a developmental transcriptional program by activating the expression of cleavage-stage genes, including genes involved in zygotic genome activation (Hendrickson et al., 2017; Iaco et al., 2017). Except for the testis and thymus, its expression is repressed in adult tissues by epigenetic silencing of the D4Z4 repeat arrays (Das and Chadwick, 2016). Facioscapulohumeral muscular dystrophy (FSHD) is caused by loss of epigenetic repression in this region, either by contraction of the D4Z4 array (FSHD type 1) or by mutations in genes involved in repression maintenance, such as *SMCHD1* (FSHD type 2), both of which lead to aberrant expression of DUX4 in skeletal muscle (Greef et al., 2009; Lemmers et al., 2010, 2012; Wijmenga et al., 1992). This disease affects approximately 870,000 people worldwide and is characterized by progressive muscle degeneration, frequently beginning with degeneration of the facial and scapular muscles and then progressing to other muscle groups. Ultimately, this leads to profound impairment of daily activities, pain and emotional distress (Schätzl et al., 2021; Statland and Tawil, 2016). How DUX4 expression causes the muscle degenerative phenotypes associated with FSHD is still an active area of investigation; however, substantial evidence suggests that activation of the DUX4 transcriptional program in skeletal muscles leads to phenotypes including oxidative stress, DNA damage, loss of proteostasis, and inflammation, which culminate in myofiber death (Bosnakovski et al., 2017; Dmitriev et al., 2016; Homma et al., 2015; Rickard et al., 2015; Shadle et al., 2017).

DUX4 is a transcription factor and, thus, most studies to date have focused on the transcriptional response to DUX4 expression (Campbell et al., 2018). Although much has been learned from these studies, understanding changes in the proteome is necessary to provide a complete picture of the cellular response to DUX4 expression. A recent study performed proteomics on DUX4-expressing myoblasts and found that there was a disconnect between RNA and protein levels; most of the RNAs that showed elevated levels did not exhibit a corresponding increase in protein levels (Jagannathan et al., 2019). Besides altering gene expression, cells can also respond to stress by changing post-translational modifications of proteins to affect their activity. To date, no studies have examined changes in post-translational modifications due to DUX4 expression at a proteome-wide level. In addition, experiments examining the DUX4-responsive transcriptome and the aforementioned proteomics dataset analyzed only a single timepoint following DUX4 expression (Geng et al., 2012, Jagannathan et al., 2016; Jagannathan et al., 2019). Thus, an understanding of the chain of events beginning with DUX4 expression and culminating in cell death is presently lacking.

To address these questions, we performed longitudinal RNA sequencing (RNAseq), proteomics and phosphoproteomics at multiple timepoints following induction of DUX4 expression in myoblasts. In agreement with previous studies, we found large-scale changes in the transcriptome without detectable corresponding changes in the proteome for most genes within 14 h following DUX4 induction. DUX4 expression also caused changes in the prevalence of splice isoforms for hundreds of mRNAs. Several cellular and molecular phenotypes that were examined manifested within 6 h of DUX4 induction. Interestingly, we also detected widespread changes in protein phosphorylation as an early event following DUX4 induction. Further exploration of the protein Jun, which displayed increased phosphorylation as determined by phosphoproteomics, led to the discovery that DUX4 expression results in the activation of two stress-responsive mitogen-activated protein kinases (MAPKs), namely, Jun N-terminal kinase (JNK) and p38, and that inhibition of these kinases ameliorated DUX4-induced cell death. We describe a role for p38 in DUX4-mediated cell death independent of its function in regulating DUX4 expression and uncover a potential positive feedback loop between p38 activity and DUX4 expression. Thus, changes in protein phosphorylation can mediate DUX4 phenotypes.

## RESULTS

### Characterization of key molecular events in inducible-DUX4 myoblasts during induction

**Fig. 1.**
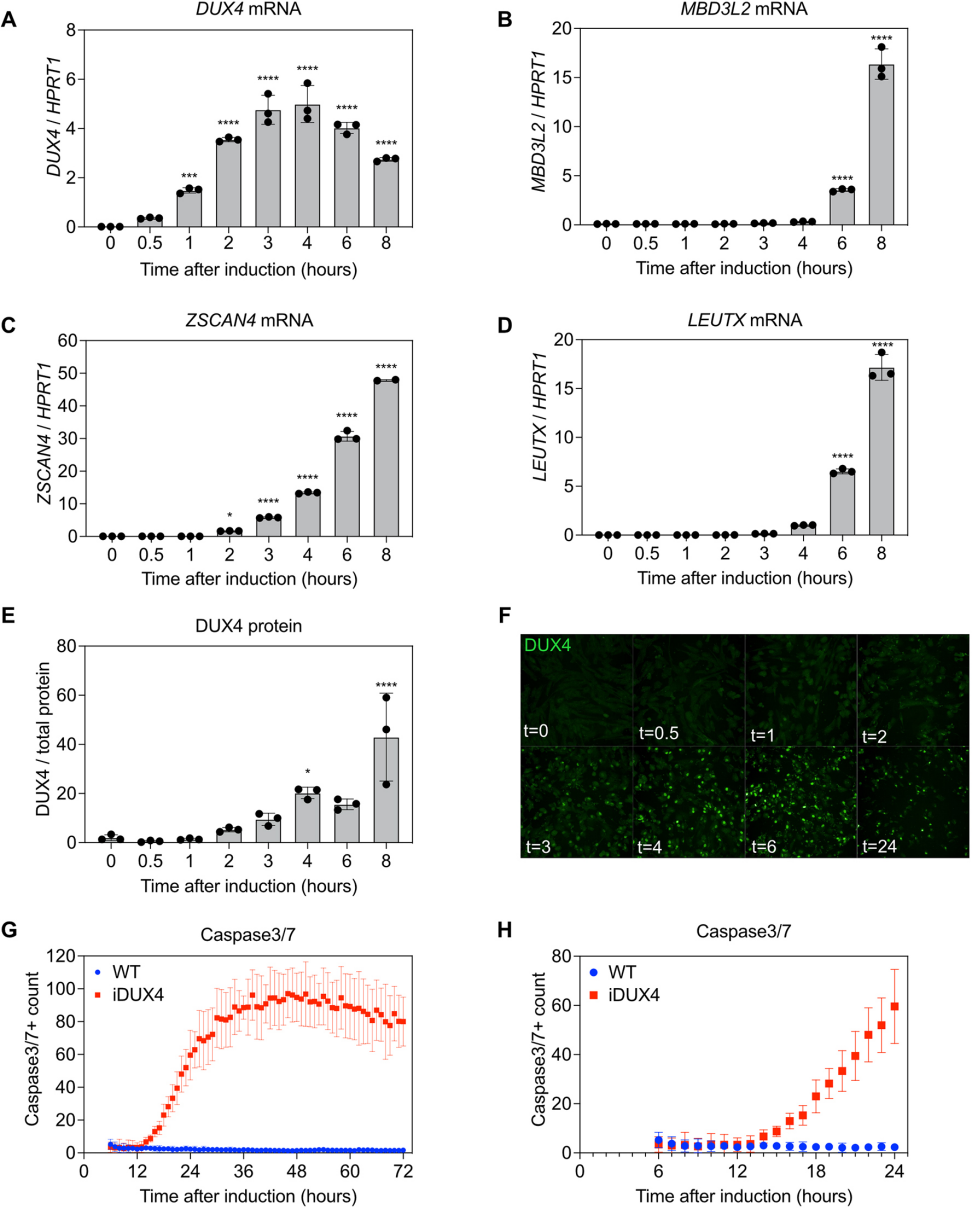
**Determining the kinetics of DUX4 induction in iDUX4 myoblasts.** In all panels, DUX4 expression in MB135 iDUX4 myoblasts was induced with 1 µg/ml doxycycline and analysis was performed at the indicated timepoints. (A-D) mRNA expression of *DUX4* (A), *MBD3L2* (B), *ZSCAN4* (C) and *LEUTX* (D) was measured using ddPCR and normalized to *HPRT1* expression. (E) DUX4 protein levels were analyzed using capillary-based western blotting and normalized to total protein levels. For A-E, error bars represent s.d. (*n*=3). *P<0.05; ***P<0.001; ****P<0.0001; one-way ANOVA with Dunnett's multiple comparisons for each timepoint compared to t=0. (F) Cells were fixed with paraformaldehyde at the indicated timepoints (in hours) and stained for DUX4. (G,H) The Caspase3/7 Dye was added simultaneously upon DUX4 induction. Cells were monitored by live-cell imaging and quantified by counting the number of cells positive for the Caspase 3/7 Dye per well. The entire 72 h time course is plotted in G and the first 24 h are shown in H. Error bars represent 95% confidence intervals. *n*=4.

Defining the exact timing of molecular events following DUX4 expression requires a system with precise control of DUX4 expression. To this end, we utilized a previously described cell model (MB135 iDUX4 myoblasts) that features DUX4 under the control of a doxycycline inducible promoter (inducible DUX4 or iDUX4) (Jagannathan et al., 2016). Induction of DUX4 using 1 µg/ml doxycycline in this model results in the expression of many of the same genes that are upregulated in FSHD patient muscle biopsies relative to healthy volunteer muscle. iDUX4 myoblasts also exhibit some of the same phenotypes observed in differentiated myotubes derived from FSHD patients, such as oxidative stress and splicing defects (Jagannathan et al., 2016). To determine the kinetics of DUX4 expression and activity, we performed a time-course experiment following induction of DUX4 with 1 µg/ml doxycycline and analyzed the mRNA levels of *DUX4* and DUX4 target genes (*MBD3L2*, *ZSCAN4* and *LEUTX*) (Geng et al., 2012) longitudinally by droplet digital PCR (ddPCR) as well as DUX4 protein levels by capillary-based western blotting and immunofluorescence (IF) imaging. Using these sensitive methods, we were able to detect *DUX4* mRNA as early as 1 h post induction with 1 µg/ml doxycycline (Fig. 1A). Interestingly, *DUX4* mRNA levels increased for the first 4 h following induction, followed by a decrease by the end of the experiment (8 h). Analysis of later timepoints revealed that *DUX4* mRNA levels increased again after 14 h of induction (Fig. S2E). This phenomenon is likely due to degradation of *DUX4* mRNA and subsequent inhibition of RNA turnover mechanisms such as nonsense-mediated decay (NMD), which has been shown previously to be associated with *DUX4* expression (Feng et al., 2015). *ZSCAN4* was the first DUX4 target gene to become activated at 2 h post induction, whereas *MBD3L2* and *LEUTX* mRNA levels were significantly increased after 6 h of induction (Fig. 1B-D). Consistent with this, an increase in DUX4 protein levels was initially noticeable 2 h post induction, indicating that this is the earliest time at which the DUX4 protein is expressed and active (Fig. 1E,F). DUX4 protein levels and the mRNA levels of DUX4 target genes were also increased throughout the 8-h experiment. To determine whether this increase in DUX4 protein was synchronous across individual myoblasts, we performed immunofluorescence staining and observed that most nuclei were faintly positive for DUX4 after 2 h of induction and that this signal increased in intensity until the end of the experiment at 8 h (Fig. 1F). We note that not all nuclei stained positively for DUX4 and there was some heterogeneity in the intensity of the DUX4 signal; however, we concluded that DUX4 induction was largely synchronous in the MB135 iDUX4 myoblasts. Importantly, the parental MB135 myoblasts that do not contain the iDUX4 transgene [hereafter referred to as wild type (WT)] did not express *DUX4* mRNA, DUX4 protein or DUX4 target genes in any of these experiments when treated with 1 µg/ml doxycycline (Fig. S1).

Finally, we performed live-cell imaging following DUX4 induction and stained the cells for Caspase 3/7, a marker of apoptosis. After 15 h of induction, the proportion of iDUX4 myoblasts that stained positively for Caspase 3/7 was significantly increased compared to WT myoblasts that had also been treated with 1 µg/ml doxycycline (Fig. 1G,H). The proportion of Caspase 3/7-positive cells continued to increase for 48 h and then gradually decreased as cells died until the end of the 72-h experiment. Thus, we conclude that the molecular events that cause DUX4-induced apoptosis begin within 15 h of DUX4 induction.

### Temporal analysis of the transcriptome and proteome following DUX4 induction

After determining the timing of DUX4 expression, activity and resultant cell death, we performed a multi-timepoint RNAseq, proteomics and phosphoproteomics analysis to generate a comprehensive understanding of the molecular events brought on by DUX4 activity, which eventually lead to cell death. For these experiments, we decided to analyze MB135 iDUX4 myoblasts at 2, 6 and 14 h post induction with 1 µg/ml doxycycline. These timepoints were selected because 2 h post induction was the earliest timepoint when DUX4 was active, the 6 h timepoint was when all analyzed DUX4 target genes were robustly expressed, and the 14 h timepoint directly preceded the onset of apoptosis. All timepoints were analyzed relative to iDUX4 lines treated with DMSO only (control) for 14 h (hereafter referred to as uninduced). We observed a low level of ‘leaky’ DUX4 expression in uninduced myoblasts, which was associated with significant changes in mRNA and protein levels of many genes in uninduced iDUX4 cells compared to uninduced WT cells (Fig. S2A,B), which is similar to what had previously been described (Karpukhina et al., 2021). As such, our analyses represent changes in gene expression relative to this low basal level of DUX4 expression. Although we chose to focus our analysis on comparisons of induced iDUX4 myoblasts to uninduced iDUX4 myoblasts, our datasets also contain comparisons of induced and uninduced iDUX4 myoblasts to WT myoblasts. Further investigations of these data might yield additional insights into DUX4-mediated alterations in the transcriptome, proteome and phosphoproteome (Tables S1 and S2). We verified that DUX4 and several known DUX4 target genes were expressed as expected in these samples (Fig. S2C-F). Additionally, doxycycline treatment had a minimal effect on RNA and protein expression; WT myoblasts treated with doxycycline for 14 h had very few significant changes in mRNA expression and no significant changes in protein levels compared to those of WT myoblasts treated with DMSO for 14 h (Fig. S2G,H).

**Fig. 2.**
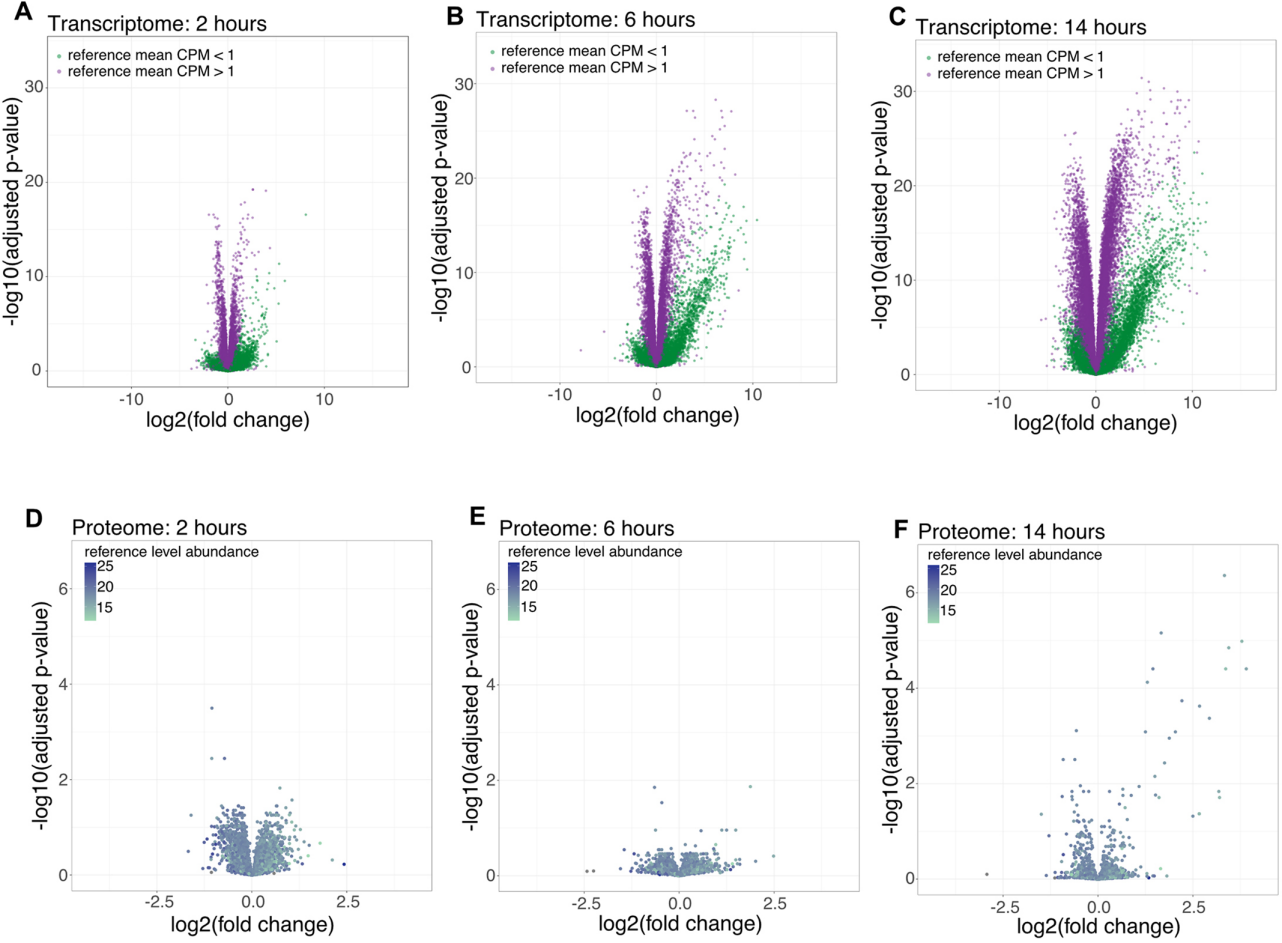
**RNAseq and proteomics following DUX4 expression.** (A-C) DUX4 expression was induced in MB135 iDUX4 myoblasts as in Fig. 1 and RNA was harvested 2 h (A), 6 h (B) and 14 h (C) after DUX4 induction for sequencing. RNAseq was also performed with iDUX4 myoblasts treated with an equivalent amount of DMSO for 14 h, which served as a reference for all timepoints. In many cases, genes that were upregulated upon DUX4 induction were undetected or very lowly expressed in uninduced cells; the voom transformation uses a pseudo count to allow for quantification resulting in genes with a normalized counts per million (CPM) value <1 in the uninduced samples (green dots) forming a distinct cluster compared to genes that were more highly expressed in the uninduced reference (purple dots). Each dot represents a gene (*n*=22,527). Data represent four biological replicates per condition. (D-F) DUX4 expression was induced as above and cell lysates were harvested 2 h (D), 6 h (E) and 14 h (F) after DUX4 induction. Samples were labeled for TMT proteomics and quantified by MS. iDUX4 myoblasts treated with an equivalent amount of DMSO for 14 h served as a reference for all timepoints. Proteins are colored according to their abundance in the reference sample. Each dot represents a unique protein (*n*=4098). Data represent three biological replicates per condition for iDUX4 samples and two biological replicates per condition for WT samples.

A comparison of uninduced and induced MB135 iDUX4 myoblasts (at 2 h post induction) showed that DUX4 expression was associated with a rapid and extensive alteration in the transcriptome, with 2.5% of the measured genes being significantly increased [false discovery rate (FDR)<0.05 and log_2_(fold change)>1] and 0.6% being significantly decreased [FDR<0.05 and log_2_(fold change)<−1] (Fig. 2A; Table S1). At 6 h post DUX4 induction, the percentage of affected genes that were significantly upregulated increased to 9.9% and those that were significantly downregulated increased to 2.7% (Fig. 2B; Table S1). At 14 h, the expression of 39% of quantified genes was significantly altered, with 24.3% being significantly upregulated and 14.6% significantly downregulated (Fig. 2C; Table S1). We then measured changes in the proteome due to DUX4 expression. After 2 h following DUX4 induction, three out of 4098 (0.1%) measured proteins were significantly upregulated [FDR<0.1, log_2_(fold change)>1] and three (0.1%) were significantly downregulated [FDR<0.1, log_2_(fold change)<−1], and protein levels were not significantly altered after 6 h (Fig. 2D,E). After 14 h of DUX4 induction, 19 proteins (0.5%) were significantly upregulated and one (0.0%) was significantly downregulated (Fig. 2F). The 19 upregulated proteins were exclusively DUX4-regulated genes (Geng et al., 2012; Young et al., 2013) (Table S2). A lack of correlation between RNA and protein levels in response to DUX4 induction has previously been described (Jagannathan et al., 2019). The authors demonstrated that DUX4 induction causes global inhibition of translation via protein kinase R (PKR)-mediated phosphorylation of the translation initiation factor eIF2α in response to the accumulation of double-stranded RNA (dsRNA) due to DUX4 activity (Jagannathan et al., 2019; Shadle et al., 2017). We compared the 14[Supplementary-material sup1]h timepoint from our dataset to RNAseq and proteomics data generated in these studies and found that the RNAseq data were well correlated (Pearson correlation r=0.923; Fig. S3A,B; Table S1). For the proteomics comparison, the levels of most proteins were unchanged upon DUX4 expression in both studies and many of the upregulated proteins were elevated in both datasets; however, some proteins that were found to be upregulated by Jagannathan et al. (2019) were not upregulated in our dataset (Pearson correlation r=0.309; Table S2). We note that the previous study used stable isotope labeling of amino acids in cell culture (SILAC), whereas our study used tandem mass tag (TMT) labeling to enable the comparison of multiple timepoints. As TMT data are known to be affected by ratio compression, this likely explains the observed differences in the two datasets (Savitski et al., 2013). In addition to translation inhibition, there are technical limitations of mass spectrometry (MS)-based proteomics compared to RNAseq, such as decreased sensitivity (Timp and Timp, 2020) and fewer biological replicates that are performed, which might contribute to a lack of correlation between mRNA and protein levels as well. It might also take additional time for the proteins encoded by these mRNAs to accumulate to levels that are detectable by MS.

### DNA damage, splicing alterations and RNA expression from intergenic regions are early events following DUX4 expression

We performed gene set enrichment analysis (GSEA) to examine the changes in mRNA levels in response to DUX4 expression to provide further insights into the cellular response to DUX4 over time. Comparative analysis of the 2-h induced and 6-h induced iDUX4 myoblasts with uninduced iDUX4 myoblasts surprisingly revealed no gene sets with significant, positive enrichment scores (Table S3). Gene sets with significant negative enrichment scores were largely related to the cell cycle. One possible explanation for this is that DUX4 affects cell cycle progression. A second explanation is that cell cycle effects are caused by less time in culture for the 2-h and 6-h timepoints compared to the reference condition (mock treatment for 14 h due to limitations on the number of samples). We favor the latter explanation as these terms are no longer significantly negatively enriched in the 14-h induced samples, indicating that this is a cell culture artifact (Table S3). After 14 h of DUX4 induction, a number of significant gene sets with positive enrichment scores were identified, including those associated with known DUX4-affected events, such as DNA damage and epigenetic regulation (Table S3).

**Fig. 3.**
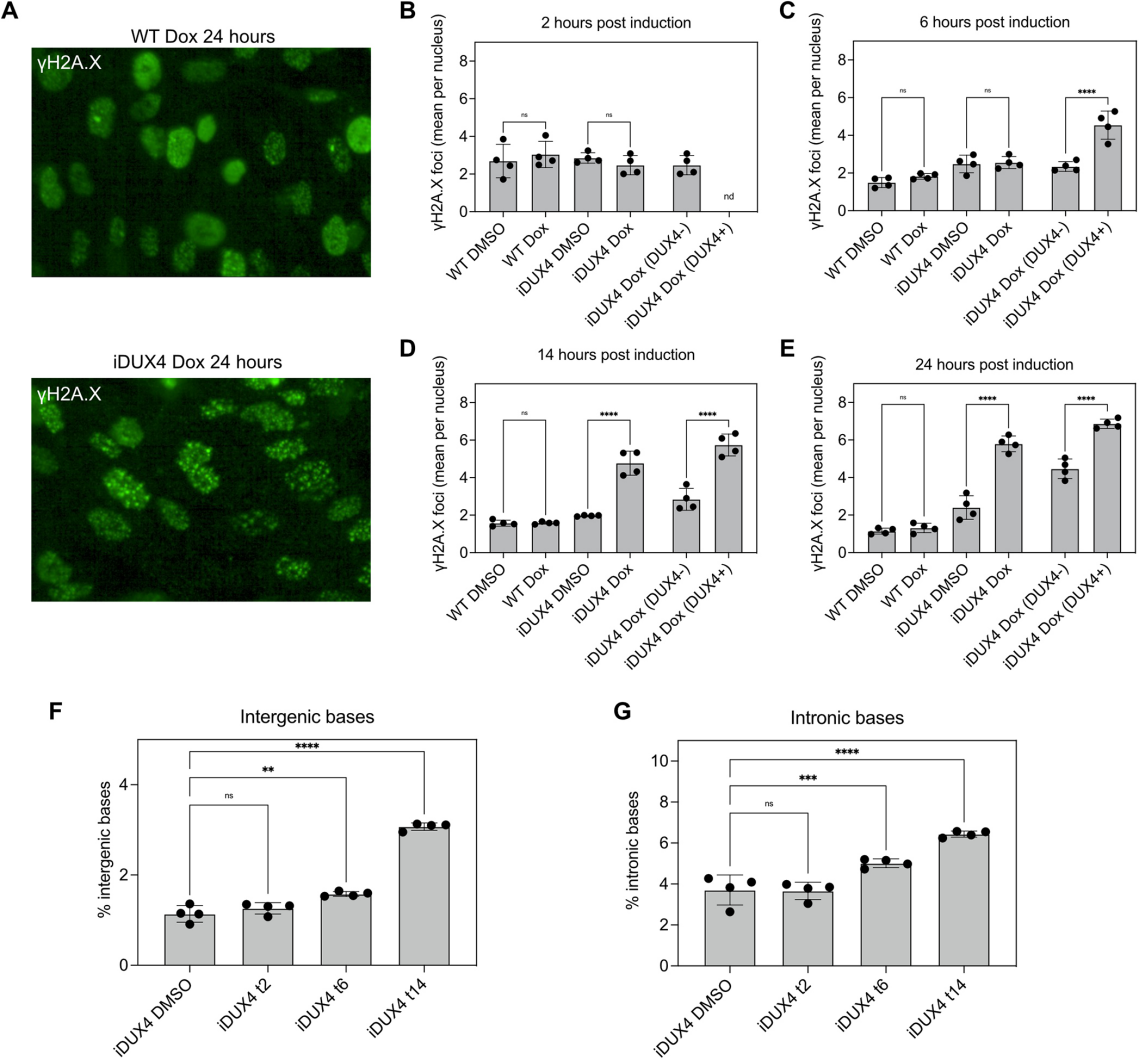
**Determination of kinetics of DUX4-mediated phenotypes.** (A-E) DUX4 expression in iDUX4 and WT myoblasts was induced as in Fig. 1. Cells were fixed at the indicated timepoints and stained for DUX4 and γH2A.X. (A) Representative image of WT and iDUX4 myoblasts induced for 24 h. (B-E) Quantification of the number of γH2A.X foci per nucleus 2 h (B), 6 h (C), 14 h (D) and 24 h (E) post DUX4 induction. The first four bars are quantifications of all nuclei in each group. The last two bars are iDUX4 myoblasts that were separated based on DUX4 expression. nd, not detected. (F,G) Percentage of RNAseq reads mapping to intergenic (F) and intronic (G) regions of the genome in uninduced and doxycycline-induced iDUX4 myoblasts. Error bars indicate s.d. *n*=4. ns, not significant; **P<0.01; ***P<0.001; ****P<0.0001; one-way ANOVA with Dunnett's multiple comparisons.

As DUX4 has previously been demonstrated to induce DNA damage (Dmitriev et al., 2016), we further explored the timing of DNA damage by monitoring the formation of γH2A.X foci, which mark sites of DNA damage (Fig. 3A) (Kuo and Yang, 2008). A comparison of induced iDUX4 myoblasts (after 2 h) to WT or uninduced iDUX4 myoblasts showed no significant increase in the number of foci per nucleus (Fig. 3B). After 6 h of DUX4 induction, there was a significant increase in DNA damage in the nuclei of cells in which DUX4 levels were high enough to be detected by IF (73.7% of all nuclei, Fig. 1F, Fig. 3C). The number of γH2A.X foci per nucleus continued to increase by 14 or 24 h after DUX4 induction (Fig. 3D,E).

We also observed an increase in reads mapping to both intergenic and intronic regions of the genome following DUX4 induction, as quantified by the Picard toolkit (https://broadinstitute.github.io/picard/) (Fig. 3F,G). One possible explanation for an increase in intergenic reads is the expression of retrotransposable elements, a well-described consequence of DUX4 expression (Geng et al., 2012; Young et al., 2013). This increased expression of intergenic elements was not apparent at 2 h post DUX4 induction but was notable at 6 and 14 h after DUX4 induction (Fig. 3F). It is possible that reads mapping to intronic regions arise either through defects in splicing or by inhibition of NMD, both of which have been shown to be associated with DUX4 expression (Feng et al., 2015; Rickard et al., 2015). Consistent with the timing of DNA damage and intergenic DNA expression, 2 h of DUX4 induction was not sufficient to cause an increase in intronic reads, but an increase was observed at the 6-h and 14-h timepoints (Fig. 3G). We conclude that DNA damage, RNA splicing alterations, NMD inhibition and retroelement expression are early responses to DUX4 induction and manifest within 6 h of DUX4 induction.

### DUX4 expression affects splice isoform prevalence

**Fig. 4.**
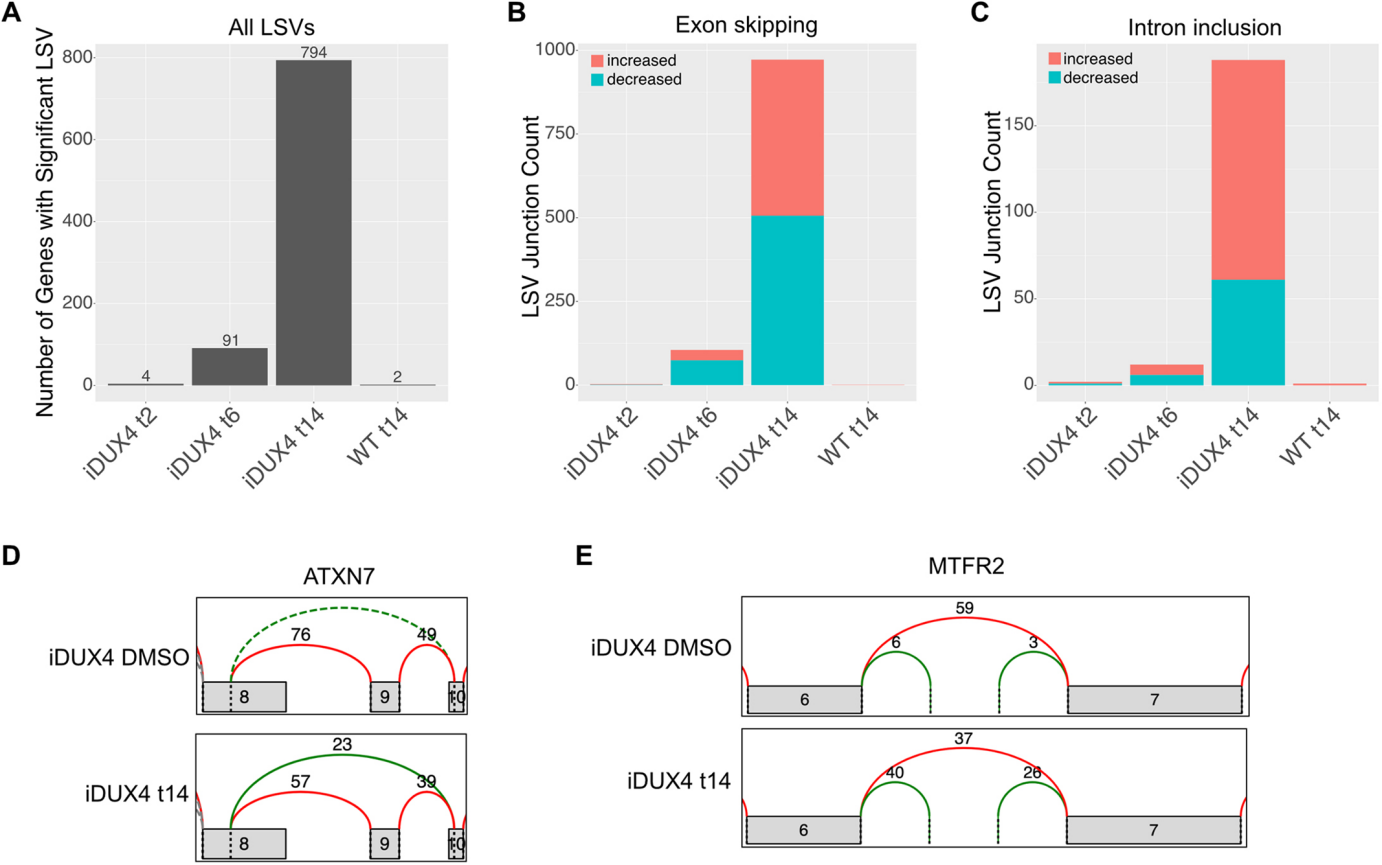
**DUX4 induces changes in splice isoforms.** (A-C) Quantification of all LSVs (A), exon-skipping events (B) and intron inclusion events (C) using MAJIQ for splice variation analysis of RNAseq data from iDUX4 myoblasts induced for 2, 6 and 14 h relative to uninduced iDUX4 myoblasts or from WT myoblasts induced for 14 h relative to uninduced WT myoblasts. LSVs were counted if they changed by 20% or more compared to the reference with 95% confidence. Red bars in B,C indicate increases in exon skipping or intron inclusion and green bars indicate decreases relative to the reference. (D,E) VOILA visualizations of examples of changes in splicing in iDUX4-induced myoblasts (bottom) compared to uninduced cells (top). Splice junctions are indicated by curved lines: red, annotated in Ensembl reference transcriptome; green, unannotated but detected in RNAseq data; dashed, undetected in the condition shown.

To determine whether alterations in splicing and inhibition of NMD resulting from DUX4 expression (Feng et al., 2015) leads to the presence of splice variants, we used Modeling Alternative Junction Inclusion Quantification (MAJIQ) to quantify changes in local splice variations (LSVs) (Vaquero-Garcia et al., 2016) [LSV analysis data available at the Dryad Digital Repository (Hill et al., 2022): https://doi.org/10.5061/dryad.k0p2ngfbh]. An LSV is defined by a specific exon and includes either all splicing and intron retention events that target that exon, or all splicing and intron retention events for which that exon is a source. Each LSV contains multiple splicing events (e.g. specific splice junctions or retained introns). A splicing event was considered significant if there was >95% confidence that the percent spliced inclusion (PSI) of that event changed by at least 20% in the induced iDUX4 cells compared to the iDUX4 DMSO-treated cells (Vaquero-Garcia et al., 2016). An LSV was considered to have a significant change if at least one splicing event in the LSV was significant. Very few splicing changes were detected at 2 h after DUX4 induction (Fig. 4A). Consistent with the timing of the increase in intronic reads, we detected 91 genes with significantly altered LSVs at 6 h after DUX4 induction. These included changes in the prevalence of retained introns and skipped exons (Fig. 4B,C). At 14 h, differential splicing events were detected in 794 genes, indicating changes in RNA species present in the cell, possibly due to splicing alterations, NMD dysfunction, or both (Fig. 4A-C). For example, in uninduced iDUX4 myoblasts, there was no evidence of skipping of *ATXN7* exon 9. However, at 14 h after DUX4 induction, reads were observed that contained an unannotated splice junction from exon 8 to exon 10, skipping exon 9 (Fig. 4D). This was accompanied by a decrease in the number of reads corresponding to the annotated splice junctions including exon 9 (Fig. 4D). As exon 9 is 260 nucleotides long, skipping of the exon is expected to introduce a frame shift relative to the annotated transcripts containing that exon. Another example of alterations in splice isoforms caused by DUX4 expression was observed with *MTFR2*. In uninduced myoblasts, an intronic region between exon 6 and exon 7 of *MTFR2* was rarely included. However, at 14 h after induction, this intronic region was observed more frequently at the expense of the canonically spliced *MTFR2* mRNA sequence (Fig. 4E). Whether these unique splice variants contribute to DUX4-mediated cellular aberrations and FSHD pathology will be of interest to study in future experiments.

### Phosphoproteomics reveal alterations in post-translational modifications due to DUX4 expression

**Fig. 5.**
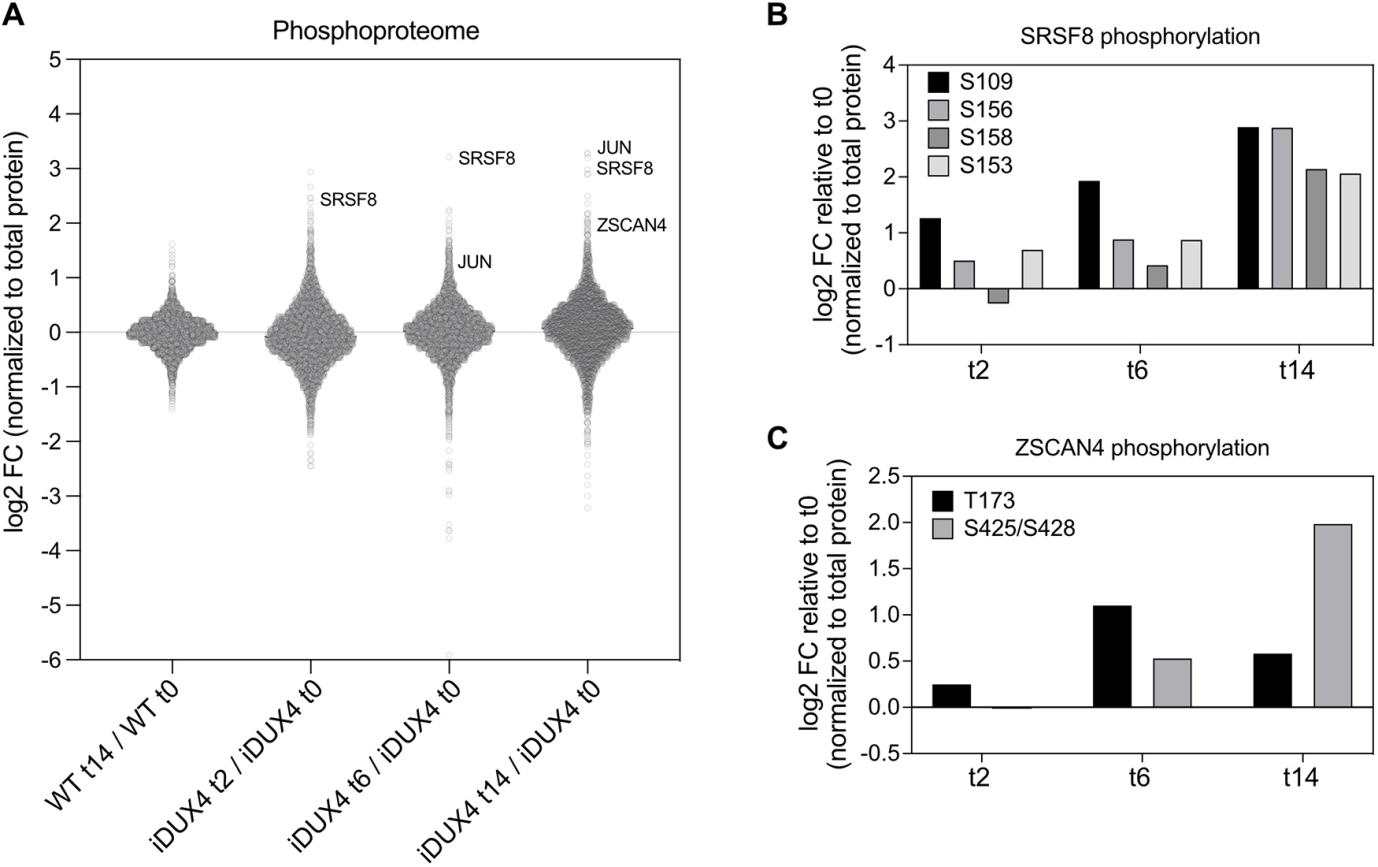
**DUX4 modulates protein phosphorylation.** (A) The phosphoproteome was quantified at the peptide level by TMT mass spectrometry and represented as a dot plot. Each phosphopeptide was normalized to total protein levels from MS (Fig. 2) to account for changes in protein levels. Each dot represents a peptide (*n*=4993) plotted as a function of its log_2_[fold change (FC)] relative to the reference. DMSO-treated iDUX4 myoblasts served as a reference for doxycycline-treated iDUX4 samples, and DMSO-treated WT myoblasts served as a reference for doxycycline-treated WT samples. Some examples of peptides with increased phosphorylation are labeled. (B,C) Examples of proteins with multiple peptides displaying increased phosphorylation on the indicated sites as determined by MS.

As there were clear differences in cell physiology despite the modest detectable changes in protein expression, we hypothesized that post-translational modifications might play a role in altering cell physiology in response to DUX4 expression. Indeed, global measurements of the phosphoproteome following DUX4 induction (after 2 h) revealed 324 peptides with at least a twofold increase or decrease in phosphorylation (Fig. 5A). Importantly, only 20 peptides exhibited an increase or decrease of phosphorylation (by more than twofold) in doxycycline-treated WT myoblasts compared to DMSO-treated WT myoblasts, indicating that the changes observed in iDUX4 cells were largely due to DUX4 induction. One protein that showed increased phosphorylation within 2 h of DUX4 induction and that continued to increase over time was the splicing factor SRSF8 (Soret et al., 1998) (Fig. 5B). Little is known about kinases acting on SRSF8 or the effects of SRSF8 phosphorylation, but it would be interesting to determine whether increased phosphorylation of SRSF8 was partially responsible for DUX4-mediated splicing defects. Of note, we also observed significant increases in phosphorylation of the DUX4 target ZSCAN4 after 6 and 14 h of DUX4 induction (Fig. 5C). These analyses were performed after normalizing to changes in total protein levels to account for any changes in protein levels caused by DUX4 expression. Like SRSF8, additional experiments are needed to understand the role that ZSCAN4 phosphorylation has on its function. We speculate that increased phosphorylation of these proteins involved in known DUX4-regulated processes might be important for mediating FSHD phenotypes; however, more information on the functional consequences of these specific phosphorylation events is needed to determine their potential disease impact.

### DUX4 expression activates stress-responsive MAPK signaling

**Fig. 6.**
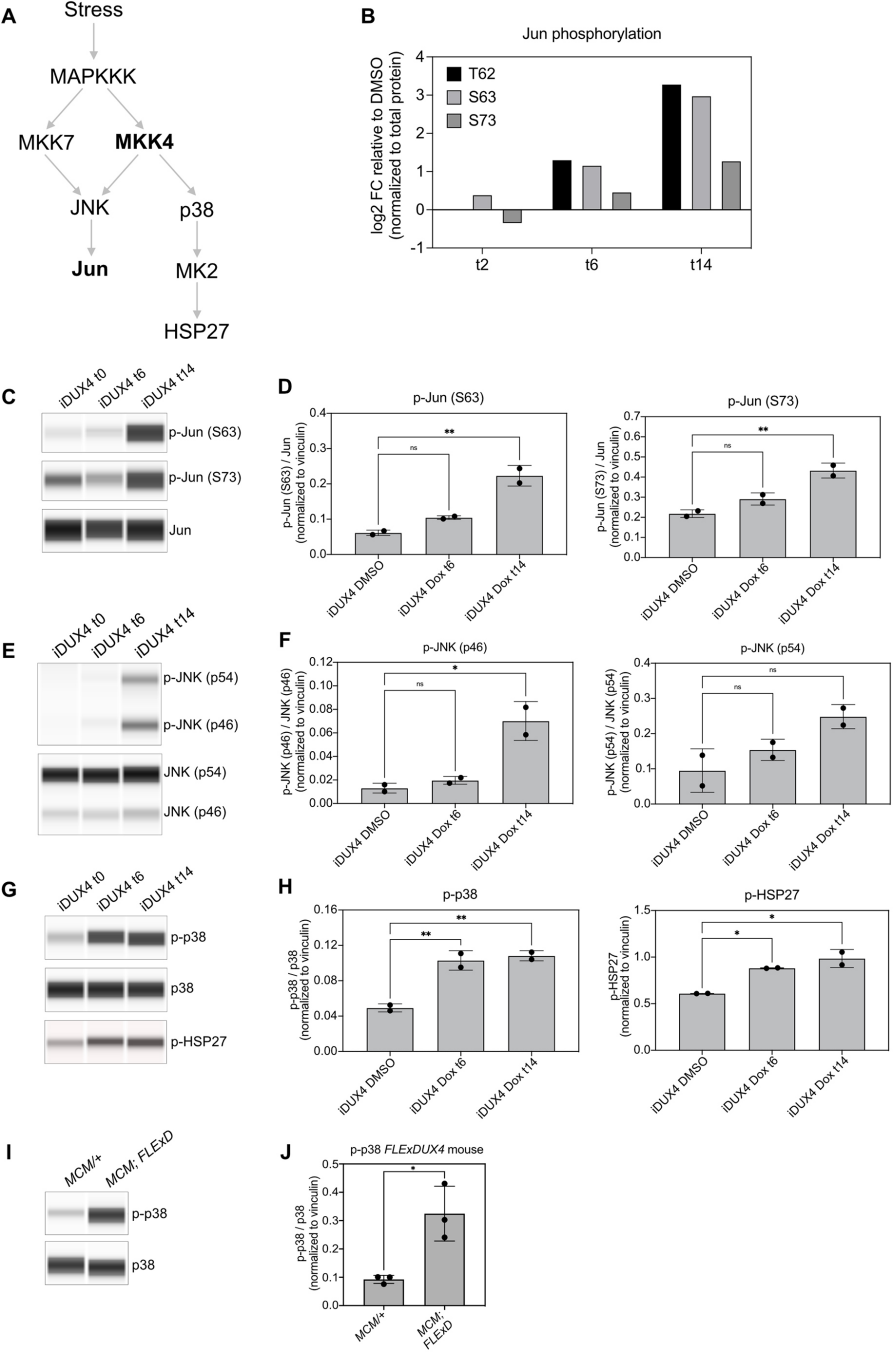
**DUX4 expression activates JNK and p38.** (A) Diagram of relevant components of the JNK and p38 MAPK signaling pathways. Multiple MAP3Ks (MAPKKK) can transmit signals from stress and other external cues to MAP2Ks. MKK7 and MKK4 are listed to illustrate cross talk between JNK and p38 signaling, although other MAP2Ks can signal through p38. Gray arrows indicate phosphorylation events. Bold text indicates proteins that we identified by proteomics. (B) Phosphorylation levels of Jun peptides as measured by MS following induction of DUX4 relative to the uninduced controls. Measurements are normalized to total protein as in Fig. 5. (C-H) Capillary-based western blotting (C,E,G) and quantification (D,F,H) of components of the JNK (C-F) and p38 (G,H) pathways in MB135 iDUX4 myoblasts. All protein levels were first normalized to vinculin levels to control for loading, then the phosphorylated proteins were normalized to their corresponding total protein to control for any changes in protein levels. Phosphorylated HSP27 was normalized to vinculin only because a commercially available antibody failed to detect total HSP27. Phosphorylation sites (C) and isoforms detected (E) are indicated in parentheses. Error bars represent s.d. *n*=2, ns, not significant; *P<0.05; **P<0.01; one-way ANOVA with Dunnett's multiple comparisons. (I,J) Capillary-based western blotting (I) and quantification (J) of p38 phosphorylation in gastrocnemius from *ACTA1-MCM/+* (*MCM/+*) and *ACTA1-MCM; FLExDUX4* (*MCM; FLExDUX4*) mice injected with 5 mg/kg tamoxifen and analyzed 9 days post injection. Normalization and quantification were performed as in D,F,H. Error bars represent s.d. *n*=3. *P<0.05; unpaired two-tailed *t*-test.

To test our hypothesis that changes in kinase signaling mediates DUX4-associated phenotypes, we explored the JNK signaling pathway (Fig. 6A). We focused on the JNK pathway because multiple peptides of the c-Jun (Jun) transcription factor, the substrate for which JNK is named, were among the most phosphorylated peptides following DUX4 induction (Fig. 6B). JNKs are members of the MAPK family of kinases, which includes ERK and p38 (Morrison, 2012). MAPKs are activated by dual phosphorylation in their activation loop by MAPK kinases (MAP2Ks), which are in turn phosphorylated by MAP2K kinases (MAP3Ks) (Keshet and Seger, 2010). A number of MAP3Ks can be activated by a variety of intracellular and extracellular stimuli, allowing MAPKs to integrate signals from multiple sources (Cuevas et al., 2007). In the case of JNK, it is known to be strongly activated by stress, such as stress associated with ultraviolet radiation and cytokines (Johnson and Nakamura, 2007). MAPKs control the cellular response to these stimuli by phosphorylating and thereby modifying the activity of their substrates, which often include other kinases and transcription factors (Cargnello and Roux, 2011). Most components of the JNK pathway were not identified in our proteomics dataset likely due to limited coverage of the proteome, although we did observe evidence of phosphorylation of Y397 of MKK4 (MAP2K4), a MAP2K that phosphorylates JNK (Fig. S4A). The cause and consequence of Y397 phosphorylation is presently unknown. We first verified DUX4-dependent phosphorylation of Jun by capillary-based western blotting using antibodies specific for phosphorylated S63 and S73, as both of these sites are JNK targets (Dérijard et al., 1994; Hibi et al., 1993). We observed a significant increase in phosphorylation of Jun at S63 and S73 after 14 h of DUX4 induction (Fig. 6C,D). Both residues also showed a modest increase in phosphorylation after 6 h of DUX4 induction relative to total Jun protein levels, although these were not statistically significant (Fig. 6C,D). We did not detect any of the JNK family kinases (JNK1, JNK2 or JNK3) in our proteomics datasets, but we detected the presence of phosphorylation in the activation loop of the kinase using an antibody against phosphorylated Thr183 and Tyr185 (Fig. 6E,F). Both the p46 and p54 isoforms of JNK had an increase in these activating phosphorylation events; however, the increase in the p54 isoform was not statistically significant (*P*=0.066, one-way ANOVA with Dunnett's multiple comparisons; Fig. 6F). We note that low sample size in these experiments limits the power of the statistical analysis; however, taken together with our MS data, we conclude that DUX4 expression results in the activation of JNK signaling.

We did not observe components of the p38 MAPK pathway in our proteomics dataset. However, MKK4 has the ability to activate both JNK and p38, and our observation that MKK4 had increased phosphorylation (Fig. S4A) prompted us to investigate this other stress-responsive MAPK pathway (Brancho et al., 2003). Like JNK, p38 is activated in response to stress through a phosphorylation cascade and phosphorylates various substrates, including other kinases such as MAPK-activated kinase 2 (MAPKAPK2 or MK2) (Cuadrado and Nebreda, 2010; Cuenda and Rousseau, 2007) (Fig. 6A). We probed for p38 activation using an antibody specific to phosphorylated Thr180 and Tyr182, which are both located in the activation loop of the p38 kinases MAPK11, MAPK12, MAPK13 and MAPK14 (Fig. 6G). Indeed, p38 phosphorylation was significantly increased by greater than twofold after 6 h of DUX4 induction, and this increase remained constant through 14 h (Fig. 6G,H). To further verify p38 activation, we measured phosphorylation of the MK2 substrate HSP27 (or HSPB1) (Larsen et al., 1997). Like p38, HSP27 phosphorylation was increased after 6 h and 14 h of DUX4 induction (Fig. 6G,H). We were unable to detect total HSP27 in our assay; therefore, we cannot exclude the possibility that this increase in phosphorylation was due to increase in total HSP27 levels. However, given that the timing coincided with p38 phosphorylation, we conclude that DUX4 expression causes activation of p38 MAPK signaling.

To explore whether MAPK activation is conserved across other models of FSHD, we analyzed MAPK activation in the *FLExDUX4* mouse model. This model utilizes a tamoxifen-inducible and skeletal muscle-specific Cre (*ACTA1-MCM*) to activate expression of full-length human DUX4 specifically in skeletal muscle (Jones and Jones, 2018). We chose to assess MAPK activation in *FLExDUX4* animals dosed with 5 mg/kg tamoxifen 9 days post injection, by which time DUX4 and DUX4-target genes are expressed, and the mice display defects in skeletal muscle function (‘moderate model’) (Jones et al., 2020) (Fig. S4B-D). We observed a significant increase in phosphorylated p38 in the gastrocnemius isolated from the *FLExDUX4* mice compared to control animals that did not possess the *FLExDUX4* transgene (Fig. 6I,J). We attempted to assess activation of the JNK pathway in these samples by probing for phosphorylation of Jun and JNK; however phosphorylated (p-) Jun [p-Jun(S63), p-Jun(S73)] and p-JNK were undetectable in lysates from mouse gastrocnemius muscle using commercially available antibodies (data not shown). More sensitive methods will be required to verify JNK activation. We conclude that p38 activation is also a consequence of DUX4 expression *in vivo* and future experiments will be required to determine whether this activation occurs directly due to DUX4 activity or due to regeneration of the muscle following DUX-induced damage.

### JNK and p38 inhibition attenuate DUX4-mediated cell death

**Fig. 7.**
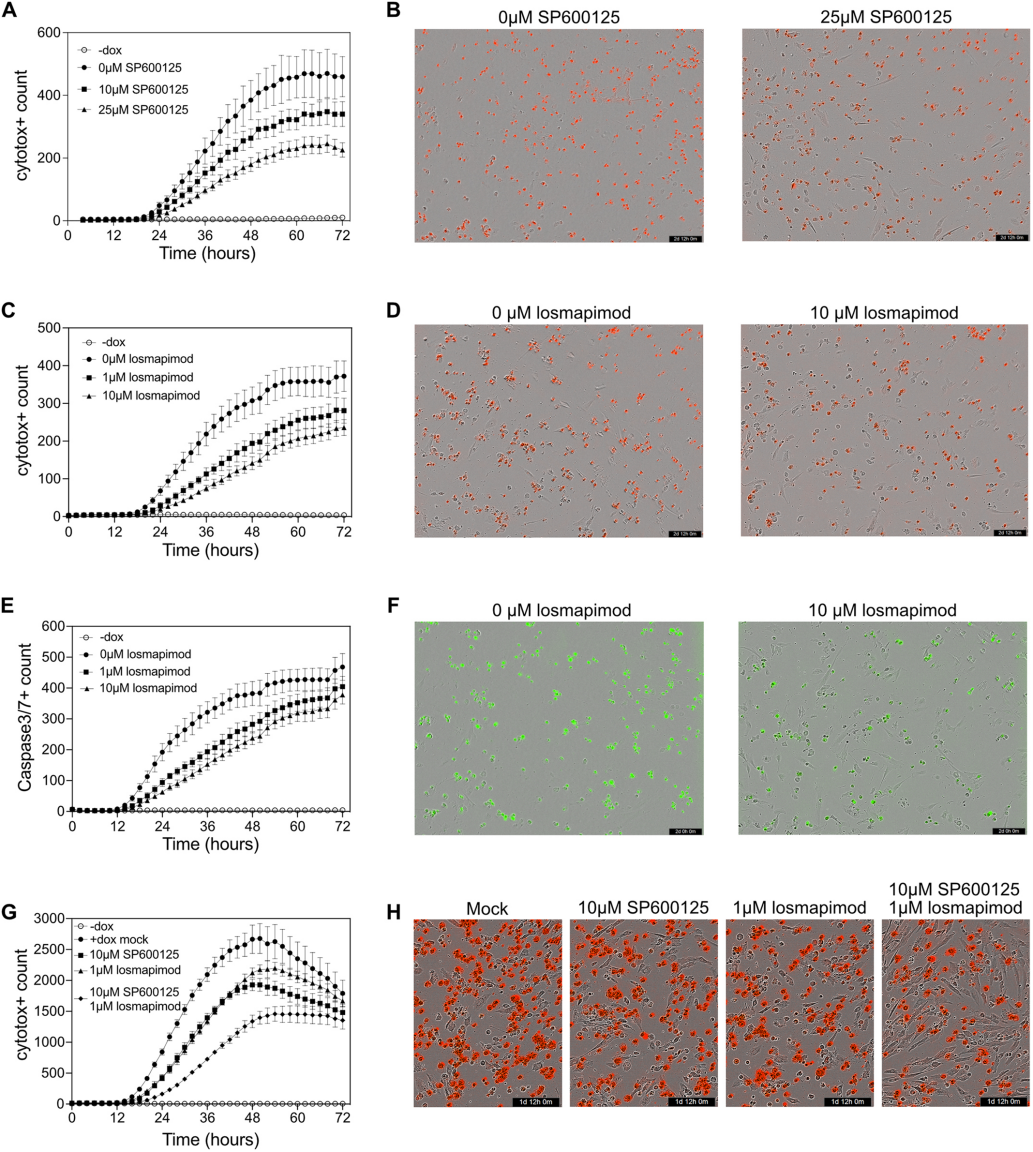
**JNK and p38 inhibition rescues DUX4-mediated cell death.** (A) DUX4 expression was induced in iDUX4 myoblasts as in Fig. 1 and cells were treated with SP600125 or vehicle and Incucyte Cytotox Dye simultaneously upon doxycycline (dox) addition. All cells received equivalent amounts of DMSO. Cells were imaged every 2 h and analyzed for dye staining. The number of cells positive for the Cytotox Dye were calculated at each timepoint. Closed symbols represent cells that received doxycycline and open circles indicate cells that did not receive doxycycline. Error bars represent 95% confidence intervals where non overlapping error bars are significantly different (*P*<0.05). *n*=8. (B) Representative images of induced iDUX4 myoblasts after 60 h either untreated (left) or treated with 25 µM SP6000125. Red spots are Cytotox-positive cells. (C-F) Live-cell imaging was performed as in A,B and cells were treated with losmapimod or vehicle and stained for either Incucyte Cytotox Dye (C,D) or Incucyte Caspase 3/7 Dye (E,F). Error bars represent 95% confidence intervals where non overlapping error bars are significantly different (*P*<0.05). *n*=8. Representative images of Cytotox-stained cells after 60 h (D) and Caspase 3/7 Dye-stained cells after 48 h (F). (G,H) Live-cell imaging was performed as in A,B and cells were treated with vehicle, SP600125, losmapimod, or both SP600125 and losmapimod. Error bars represent 95% confidence intervals where non overlapping error bars are significantly different (*P*<0.05). *n*=8. (H) Representative images of Cytotox-stained cells after 36 h.

Finally, owing to the role of JNK signaling in apoptosis, we sought to determine whether JNK activation could be responsible for inducing cell death in response to DUX4 expression. We used the JNK inhibitor SP600125 that has a reported ∼20-fold selectivity over other kinases and a 300-fold selectivity over the related MAPKs ERK and p38 (Bennett et al., 2001). SP600125 was shown to inhibit phosphorylation of Jun in cell-based assays at concentrations of 10 µM or higher (Bennett et al., 2001). We performed live-cell imaging of MB135 iDUX4 myoblasts in both the induced and uninduced states and following incubation with 10 µM or 25 µM SP600125. All cells were treated with an equivalent amount of DMSO and analyzed using a cell viability dye. Importantly, addition of SP6000125 did not affect DUX4 induction by doxycycline addition (Fig. S5A,B). Cells that were not induced with doxycycline did not undergo cell death throughout the course of the experiment regardless of whether they received SP600125, indicating a lack of general toxicity (Fig. S5C). Induction of DUX4 expression with doxycycline resulted in cell death as expected, and treatment with both 10 µM and 25 µM SP600125 reduced the degree of cell death throughout the time course in a dose-dependent manner (Fig. 7A,B). We were unable to examine Caspase3/7 activation in this assay due to green autofluorescence of the SP600125 compound (data not shown). Taken together, we conclude that JNK activation is a consequence of DUX4 expression and that this activation can contribute to DUX4-mediated cell death.

As p38 signaling is also capable of inducing apoptosis, we asked whether p38 inhibition could also prevent DUX4-mediated cell death. We chose to test the p38α/β inhibitor losmapimod because it is currently being investigated as a treatment for FSHD in a clinical trial (NCT04003974). p38 inhibition has been shown to prevent DUX4 expression in FSHD patient-derived myotubes, thereby preventing cell death (Oliva et al., 2019; Rojas et al., 2020); however, a role in DUX4 mediated-apoptosis independent of DUX4 expression has not been explored. As expected, losmapimod treatment did not affect doxycycline-induced DUX4 expression (Fig. S5D,E). We performed a similar live-cell imaging experiment as above, treating cells with mock, 1 µM or 10 µM losmapimod at the same time as doxycycline induction. Losmapimod treatment of uninduced iDUX4 myoblasts had no effect on cell death, indicating a lack of toxicity at these concentrations (Fig. S5F). Remarkably, losmapimod treatment prevented DUX4-mediated cell death in a dose-dependent manner, as demonstrated by both the viability stain (Fig. 7C,D) and the Caspase 3/7 stain (Fig. 7E,F). We conclude that p38 is activated by DUX4 expression and contributes to DUX4-dependent apoptosis, independent of its role in controlling DUX4 expression. Interestingly, simultaneous addition of 10 µM SP600125 and 1 µM losmapimod displayed decreased DUX4-mediated cell death compared to either inhibitor alone (Fig. 7G,H). This suggests that the combined inhibition of JNK and p38 signaling confers enhanced protection against DUX4 cytotoxicity.

## DISCUSSION

In this study, we sought to provide a comprehensive picture of the timing of molecular events following DUX4 expression by performing RNAseq, proteomics and phosphoproteomics at multiple timepoints following DUX4 induction. It has been previously reported that DUX4 expression causes pervasive changes in the transcriptome with few detectable changes at the protein level within 14 h following DUX4 induction (Jagannathan et al., 2019). Interestingly, we observed that several phenotypes, namely, DNA damage and splicing defects, manifest before there are detectable changes in the proteome. Although we cannot rule out the possibility that there were proteins that had altered levels at these times, which we did not observe by MS; this observation led us to examine whether changes in protein phosphorylation could explain some of the DUX4-mediated phenotypes. In support of this, we observed hundreds of proteins with both increased and decreased phosphorylation as soon as 2 h after DUX4 was induced, the first timepoint at which we were able to detect DUX4 activity. Thus, it is possible that kinase signaling networks are the first to respond to DUX4 expression.

Indeed, further exploration of one hit in this dataset (Jun) led to the discovery of the MAPKs JNK and p38 as mediators of DUX4-induced cell death. It is currently unclear what specific stimuli induce JNK or p38 activation in the context of DUX4 expression but, given that we observed p38 activation earlier than JNK activation, we speculate that they might be controlled by different mechanisms. JNK activation peaked later in our time course and, as such, it is possible that a downstream consequence of DUX4 expression (e.g. oxidative stress or DNA damage) causes activation of the pathway. Indeed, JNK is known to be activated by oxidative stress (Goldman et al., 2004; Tobiume et al., 2001). Determining what MAP3Ks in the JNK and p38 pathways are activated upon DUX4 expression might help illuminate what signal(s) are being sensed. Future experiments using genetic knockdown of specific components of the JNK and p38 pathways will shed light on this question.

In addition, it would be interesting to determine which JNK and p38 substrates are directly responsible for DUX4-mediated cell death. For example, DUX4-induced apoptosis has been reported to be p53 dependent (Wallace et al., 2010) and both JNK and p38 are capable of phosphorylating p53 (Fuchs et al., 1998; Perfettini et al., 2005). We speculate that one possible mechanism of p53 activation in these contexts could be phosphorylation by JNK or p38. Further experiments would be required to test this hypothesis.

Although JNK inhibition did prevent DUX4-mediated cell death, substantial barriers remain before JNK can be considered as a viable target for the treatment of FSHD. First, inhibition of JNK has significant safety concerns owing to its ubiquitous expression profile. There are three forms of JNK in humans: JNK1 and JNK2 are expressed widely across tissue types, whereas JNK3 expression is more restricted to the brain, heart and testes (Bogoyevitch et al., 2004). Loss of JNK1 or JNK2 is embryonic lethal in mice, indicating they have an essential role in development (Kuan et al., 1999); although, this might not be a concern for treating adults with JNK inhibitors. Beyond development, JNK signaling has been implicated in T-cell function (Dong et al., 2000) and viral activation of the innate immune response (Chu et al., 1999). Thus, inhibition of JNK in adults could result in an undesirable loss of immune responses. As JNK has also been demonstrated to be a tumor suppressor in some contexts (Teng et al., 1997), it remains possible that JNK inhibition could cause an increased risk of cancer. Although sustained JNK activation is pro-apoptotic, transient JNK activation promotes cell survival in some contexts, highlighting the complexity of this pathway (Dhanasekaran and Reddy, 2008). Thus, JNK inhibition has been explored more widely as a strategy for treating cancer as it can promote cell survival in the context of cancer (Messoussi et al., 2014; Wu et al., 2020). The pro-apoptotic functions of JNKs have been implicated in the pathology of several diseases, including Alzheimer's disease and type 2 diabetes, and, as such, JNK inhibition has been proposed as a treatment for these conditions (Bogoyevitch et al., 2004; Manning and Davis, 2003). Several JNK inhibitors have entered clinical trials; however, most of these studies were discontinued, in some cases due to poor benefit/risk profiles (Sabapathy, 2012). Finally, JNK activity is increased in skeletal muscle in response to exercise and contributes to myofiber growth (Boppart et al., 1999; Lessard et al., 2018), and thus long term JNK inhibition might be problematic for muscle regeneration, particularly in people with muscular dystrophy. However, a recent study suggested that JNK is also involved in muscle degeneration in a cancer-associated cachexia mouse model and that JNK inhibition could prevent muscle wasting (Mulder et al., 2020), providing some hope that JNK inhibition could benefit people with FSHD if toxicity in muscle and other tissues can be avoided.

In contrast to JNK, p38 inhibition is already being explored as a therapy for FSHD (Brennan et al., 2021). p38 inhibitors were found to prevent expression of DUX4 from its native locus in both FSHD patient-derived myotubes and mouse xenograft models of FSHD, paving the road for clinical development (Oliva et al., 2019; Rojas et al., 2020). The proposed mechanism of action for p38 inhibitors in FSHD is to prevent DUX4 expression; however, our data incite the possibility that prevention of apoptosis independent of DUX4 expression might be an additional mechanism by which p38 inhibition can improve muscle pathology in FSHD patients. Furthermore, we found that DUX4 expression promotes p38 activation. In conjunction with previous studies demonstrating that p38 activity is required for DUX4 expression, this might result in a positive feedback loop wherein active p38 promotes DUX4 expression and DUX4 further activates p38. More experiments will be required to test this model, which might enhance our understanding of the mechanism of p38 inhibitors in FSHD patients.

Our phosphoproteomics dataset revealed that the splicing factor SRSF8, a member of the SR family of splicing proteins, which are important for pre-mRNA splicing and alternative splicing (Long and Caceres, 2008), had multiple residues with increased phosphorylation at 2, 6 and 14 h following DUX4 induction. As splicing defects were observed at 6 h, before any noticeable changes in the proteome, it is possible that changes in post-translational modifications of splicing factors such as SRSF8 could explain alterations in splicing. In the case of SRSF8, studies to mutate phosphorylation sites that either prevent or mimic phosphorylation might shed light on their role in regulating splicing.

To our knowledge, our phosphoproteomics dataset represents the first global analysis of changes in post-translational modifications caused by DUX4 expression. Although we focused on a few proteins that showed an increase in phosphorylation, we anticipate that there might be other changes in phosphorylation that have phenotypic consequences similar to what we evaluated for JNK and p38. For example, there were many proteins that had decreases in phosphorylation after DUX4 expression. These could be due to activation of phosphatases or deactivation of kinases. We hope that future studies will examine more of these changes in depth. Moreover, as DUX4 causes alterations in the phosphoproteome, it would be interesting to determine whether other post-translational modifications are affected as well. Ubiquitination might be of interest given the perturbations in protein homeostasis brought on by DUX4 expression (Homma et al., 2015).

The MB135 iDUX4 myoblast model is well suited to assess changes in protein phosphorylation caused by DUX4 activity because of the synchronous induction of DUX4; however, there are key differences between this model and skeletal muscles of FSHD patients. First, DUX4 is thought to be expressed in myonuclei of mature muscle fibers, which might have a different set of active kinases than proliferating myoblasts. To bridge this gap, we confirmed p38 activation *in vivo* by analyzing the *FLExDUX4* mouse model. This suggests that p38 activation is a consequence of DUX4 expression in both myoblasts and mature muscle tissue. We note that the *in vivo* analyses were performed on skeletal muscle tissue isolated several days after the induction of DUX4 expression, raising the possibility that p38 activation is an indirect response of DUX4 expression *in vivo*. Indeed, p38 is known to be activated during muscle regeneration (Zetser et al., 1999). Thus, it is possible that there are separate mechanisms for DUX4-mediated p38 activation *in vitro* and *in vivo*. Future studies will be required to determine whether DUX4 directly activates p38 or whether DUX4-mediated damage to the tissue and subsequent regeneration activates p38. In either case, the finding that DUX4 expression leads to p38 activity is important to our understanding of FSHD and ongoing clinical trials. To determine whether increases in protein phosphorylation *in vivo* is the case more generally, phosphoproteomic analysis of skeletal muscle from these mice would be required to confirm this; however, there are likely species-specific differences that would limit comparison to the dataset generated in this study.

Second, DUX4 is thought to be expressed in a small subset of myonuclei in FSHD patients, whereas the majority of MB135 iDUX4 myoblasts express DUX4 (Fig. 1F). The rare, stochastic nature of DUX4 expression is recapitulated in cell culture of *in vitro* differentiated patient-derived myotubes. As most nuclei in these models do not express DUX4, bulk assays such as phosphoproteomics and western blotting would likely fail to capture DUX4-dependent differences in protein phosphorylation between FSHD myotubes and healthy myotubes. We speculate that the development of assays capable of measuring changes in protein phosphorylation at a single-nucleus resolution would enable confirmation of the observations reported in this study by specifically examining DUX4-expressing nuclei in patient-derived myotubes.

Finally, we were able to quantify changes in transcript splicing using RNAseq reads that spanned splice junctions (Vaquero-Garcia et al., 2016). We observed nearly 800 genes that had changes of at least 20% in splice junction usage or intron retention after 14 h of DUX4 induction. Thus, not only does DUX4 alter what genes are being expressed, it also changes what splice isoforms are present in myoblasts. The source of these transcripts could be a result of increases in splicing errors, decrease in turnover by NMD, or both. Further, it would be of interest to evaluate whether these unique splice isoforms encode proteins with altered functions and whether they play a role in DUX4-mediated phenotypes.

In conclusion, our longitudinal multi-omics approach led to the discovery that DUX4 expression in myoblasts causes global changes in protein phosphorylation. One mechanism by which this occurs is the activation of the JNK and p38 MAPKs, both potential therapeutic targets for FSHD. Further exploration of changes in kinase signaling in response to DUX4 expression using phosphoproteomics data might reveal additional potential targets for FSHD therapies.

## MATERIALS AND METHODS

### Cell culture

MB135 parental and MB135 iDUX4 myoblasts received from the laboratory of Stephen Tapscott (Fred Hutchinson Cancer Center, Seattle, WA, USA) were cultured as previously described (Jagannathan et al., 2016) and confirmed to be free of contamination. Briefly, cells were grown in Ham's F10 Nutrient Mix (Gibco) containing 15% fetal bovine serum (Gibco), 10 ng/ml rhFGF (Promega) and 1 µM dexamethasone (Sigma-Aldrich). To ensure presence of the DUX4-inducible cassette, 3 µg/ml puromycin (Gibco) was included in MB135 iDUX4 culture medium. Cells were passaged when they reached ∼70% confluency. Cells were allowed to attach overnight before DUX4 expression was induced by the addition of 1 µg/ml doxycycline hydrochloride (Sigma-Aldrich) and analyzed by downstream assays.

### *FLExDUX4* mice and tamoxifen injections

*ACTA1-MCM* control mice (JAX 025750) and *FLExDUX4* mice (JAX 028710) were acquired from Jackson Laboratory and were previously described (Jones and Jones, 2018). Studies were performed on 16- to 18-week-old male mice. Aliquots of tamoxifen (T5648, Millipore-Sigma) were made by first creating a 200 mg/ml solution in DMSO. This solution was stirred for several hours at room temperature (RT) while covered. Then, corn oil (Sigma-Aldrich) was added at 1:10 for a final concentration of 20 mg/ml and the solution was stirred overnight at RT while covered. Aliquots were frozen at −20°C and were diluted further with corn oil prior to dosing. Animals were dosed intraperitoneally with 5 mg/kg of tamoxifen.

All activities involving animals were carried out in accordance with federal, state, local and institutional guidelines governing the use of laboratory animals in research in an AAALAC-accredited facility following the Guide for the Care and Use of Laboratory Animals and were reviewed and approved by Pfizer's Institutional Animal Care and Use Committee.

### ddPCR

RNA was harvested using a RNeasy mini kit (QIAGEN) and on-column DNA digestion (QIAGEN) according to the manufacturer's protocol. cDNA was generated from 500 ng of RNA using SuperScript VILO (Invitrogen) according to the manufacturer's protocol. cDNA reactions were diluted 1:100 before mixing with 2× ddPCR Supermix for probes (without dUTP) (Bio-Rad) and the following Taqman assays (Thermo Fisher Scientific): *MBD3L2*, Hs00544743_m1; *ZSCAN4*, Hs00537549_m1; *LEUTX*, Hs01028718_m1; and *HPRT1*, Hs99999909_m1. A custom Taqman assay (Thermo Fisher Scientific) for codon-altered *DUX4* used the following: Primer 1, 5′-GAGAGTGCAGATCTGGTTC-3′; Primer 2, 5′-GTATTCGTGATTCTGGGAGC-3′; and probe, 5′-TCGAGAAAGATCGGTTCCCC-3′. Droplets were generated using an Automatic Droplet Generator (Bio-Rad) and 40 cycles of PCR were performed with an annealing/extension temperature of 60°C for 1 min. Droplets were then scanned using a QX200 droplet reader (Bio-Rad). Expression was quantified using QuantaSoft software (Bio-Rad) and expressed as a ratio to *HPRT1*.

For mouse studies, frozen tibialis anterior (TA) muscles were crushed in a Cryoprep Pulverizer (Covaris). Powdered tissue was added to a 2 ml microtube along with 5 mm stainless steel beads (QIAGEN) and 1 ml Trizol (Invitrogen). The tube was then homogenized for 5 min in a Tissuelyzer II (QIAGEN) at a frequency setting of 25 Hz. The liquid was transferred to a phasemaker tube (Invitrogen) and 1:5 chloroform was added. The tube was vortexed thoroughly and incubated at RT for 5 min. The tube was then centrifuged for 5 min at 14,000 ***g*** and the clear supernatant was transferred to a new microtube. From here, a Purelink RNA mini kit (Thermo Fisher Scientific) was used to collect the RNA. ddPCR was performed as above using the following Taqman assays (Thermo Fisher): *Trfc*, Mm00441941_m1; *Trim36*, Mm00619032_m1; and *Wfdc3*; Mm01243777_m1. A custom Taqman assay (Thermo Fisher) for *DUX4* used the following: Primer 1, 5′-CCCGGCTGACGTGCAA-3′; Primer 2, 5′-AGCCAGAATTTCACGGAAGAAC-3′; and probe, 5′-AGCTCGCTGGCCTCTCTGTGCC-3′. Expression was quantified using QuantaSoft software (Bio-Rad) and expressed as a ratio to *Trfc*.

### Capillary-based western blotting

Proteins were isolated using RIPA lysis buffer (Cell Signaling Technology) containing 1× Protease inhibitor (Roche) and 1 µM phenylmethylsulfonyl fluoride (PMSF). Cells were lifted from plates by scraping and allowed to lyse on ice for 30 min. Lysates were cleared by centrifugation at 11,000 ***g*** for 30 min. Capillary-based western blotting was carried out using the Simple Western system (Wes and Jess; Protein Simple) and analyzed using the Compass for Simple Western software (Protein Simple). For Fig. 1E, samples were run using the Protein Normalization Assay Module (Protein Simple, AM-PN01) and DUX4 levels were quantified relative to total protein. For Fig. 6 and Fig. S5, samples were run using the 12-230 kDa Separation Module (Protein Simple, SM-W004) and quantified relative to vinculin. The following primary antibodies were used in this study: anti-DUX4 E5-5 (Abcam, ab124699), anti-Jun (Cell Signaling Technology, 9165), anti-p-Jun(S63) (Cell Signaling Technology, 91952), anti-p-Jun(S73) (Cell Signaling Technology, 3270), anti-JNK (Cell Signaling Technology, 9252), anti-p-JNK (Cell Signaling Technology, 4668), anti-p38 (Cell Signaling Technology, 9212), anti-p-p38 (Cell Signaling Technology, 9211), anti-p-HSP27 (Cell Signaling Technology, 2401) and anti-vinculin (Millipore Sigma, SAB4200080). All primary antibodies except anti-vinculin and anti-p-HSP27 were diluted 1:50 and detected using the anti-rabbit detection module (Protein Simple, DM-001). Anti-p-HSP27 was diluted 1:10 and detected using the anti-rabbit detection module (Protein Simple, DM-001). Anti-vinculin was diluted 1:100 and detected using the anti-mouse near infrared detection module (Protein Simple, DM-009).

For mouse studies, frozen gastrocnemius muscles were crushed in a Cryoprep Pulverizer (Covaris). Powdered tissue was added to a 2 ml microtube along with 5 mm stainless steel beads (QIAGEN) and 100 µl RIPA buffer (Cell Signaling Technology) containing 1× protease inhibitor and 1× phosphatase inhibitor (Roche). The tube was then homogenized twice for 5 min in a Tissuelyzer II (QIAGEN) at a frequency setting of 25 Hz. Samples were placed on ice for 30 min, then lysates were cleared by centrifugation at 11,000 ***g*** for 30 min. Samples were analyzed using capillary-based western blotting as described above using the 12-230 kDa Separation Module (Protein Simple, SM-W004) and quantified relative to vinculin. The primary antibodies used were anti-p38 (Cell Signaling Technology, 9212), anti-p-p38 (Cell Signaling Technology, 9211) and anti-vinculin (Millipore Sigma, SAB4200080).

### Immunofluorescence

Cells were cultured in black-walled, 96-well plates (CellCarrier Ultra) and fixed by adding paraformaldehyde (Electron Microscopy Sciences) directly into the medium at a final concentration of 4% for 15 min. Cells were washed three times with PBS, then blocked with PBS containing 5% normal goat serum (Cell Signaling Technology) and 0.3% Triton X-100 for 1 to 2 h. Primary antibodies were diluted in PBS containing 1% bovine serum albumin and 0.3% Triton X-100 and incubated with cells overnight at 4°C. Cells were then washed three times with PBS. Secondary antibodies were diluted in PBS containing 1% bovine serum albumin and 0.3% Triton X-100 and incubated with cells for 2 h at ambient temperature. DNA was stained by incubating wells with Hoechst 33342 (Invitrogen) diluted 1:5000 in PBS, then washed three times with PBS prior to imaging. Images were acquired using an Opera Phenix High Content Screening System (Perkin Elmer) and quantified using Harmony software (Perkin Elmer). DUX4 was detected using the E5-5 antibody diluted 1:2500 (Abcam, ab124699) and goat anti-rabbit Alexa Fluor 488 (Invitrogen, A32731) diluted 1:1000. The primary antibody for γH2A.X (Cell Signaling Technology, 9718) was diluted 1:2500 and detected using goat anti-rabbit Alexa Fluor 488 diluted 1:1000.

### Live-cell imaging

Cells were cultured in black-walled, 96 well plates (CellCarrier Ultra) and SP600125 (MedChemExpress) or losmapimod (Selleck Chemicals) or DMSO (vehicle control) was added along with Incucyte Cytotox Dye (Sartorius) and Incucyte Caspase 3/7 Dye (Sartorius) simultaneously upon induction of DUX4. Cells were imaged every hour (Fig. 1H) or every 2 h (Fig. 7) using the IncuCyte live-cell imaging system (Sartorius). Images were analyzed using Incucyte software to quantify Cytotox- and Caspase3/7-positive cells.

### RNAseq

RNA was isolated using an RNeasy Mini Kit (QIAGEN) as above. Total RNA from 24 samples was evaluated for quality (RNA integrity number scores≥9) by 4200 TapeStation System (Agilent, Santa Clara, CA, USA) using RNA ScreenTape (5067-5576, Agilent). The concentrations were measured by High Lunatic plate (7012000, Unchained Labs, Pleasanton, CA, USA). Approximately 200 ng total RNA was used as input for the mRNASeq library preparation using the TruSeq Stranded mRNA Library Prep Kit (20020594, Illumina, San Diego, CA, USA) on the Janus G3 Automated Liquid Handler System (Perkin Elmer).

The protocol includes purification of the poly-A-containing mRNA using poly-dT beads, fragmentation of RNA, first strand and second strand cDNA synthesis, end repairing, A-tailing, adapter ligation, followed by PCR amplification to generate the final cDNA library. The final libraries were then evaluated for quality by 4200 TapeStation System (Agilent) using D1000 ScreenTape (5067-5582, Agilent). The concentrations were measured using Qubit 1× dsDNA HS Assay Kit (Q33231, Invitrogen-Life Technologies). The libraries were then normalized to 4 nM, re-quantitated using Qubit 1× dsDNA HS Assay Kit and then pooled for sequencing. The pool was further denatured and diluted to 1.8 pM and loaded on a NextSeq 500 sequencer for 75 paired-end runs using 150 cycle High Output Sequencing Kit (20024907, Illumina).

### Quantification and differential expression analysis

Libraries were demultiplexed using bcl2fastq (Illumina). Reads were aligned to the genome (GRCh38) using STAR 2.7.3a (Dobin et al., 2013). Salmon 0.14.2 (Patro et al., 2017) was used to assign aligned reads to transcripts (Ensembl version 98). Quality control metrics for reads and alignments were found using fastqc (version 0.11.8, https://www.bioinformatics.babraham.ac.uk/projects/fastqc/) and Picard Tools (version 2.18.15, https://broadinstitute.github.io/picard/), respectively, followed by summarization with multiqc (version 1.8, https://multiqc.info/). In order to quantify transcripts from the codon-altered inducible DUX4 (caDUX4), the reads were then re-aligned to a modified genome that included the caDUX4 gene sequence (Jagannathan et al., 2016). Transcripts were also re-quantified with Salmon using a modified transcriptome that also included the caDUX4 sequence.

Statistical analysis was performed in R 4.0.3. Transcripts were summarized to the gene level using tximport (version 1.16.1) (Soneson et al., 2015) with countsFromAbundance=‘lengthScaledTPM’ to account for different transcript length distributions across samples. Low-count genes were removed if they did not have at least two counts in at least four samples (the number of samples in each condition). A low threshold was selected because many known DUX4 response genes were not expected to be present in the WT or uninduced samples. Counts were normalized across samples using the relative log expression method from edgeR (version 3.30.3) (Anders and Huber, 2010). Normalized counts were transformed with voom (Law et al., 2014), and limma (version 3.44.3, Ritchie et al., 2015; https://bioconductor.org/packages/release/bioc/html/limma.html; Phipson et al., 2016) was used to fit a model of transformed counts, which was specified in the limma command as ‘∼1+ condition’, where condition corresponded to both the cell line and the treatment (DMSO or doxycycline), and to calculate log_2_(fold change), *P*-values and Benjamini–Hochberg false discovery rates for the contrasts of interest.

### Splicing analysis

Both annotated and novel splice events were quantified from the BAM file outputs from STAR using MAJIQ 2.1 (Vaquero-Garcia et al., 2016) and visualized using VOILA 2.0.0 (Vaquero-Garcia et al., 2016). The output from VOILA was parsed using a custom R script to quantify transcriptome-wide levels of intron retention and exon-skipping events, and a threshold of 95% posterior probability of ≥20% change in percent spliced inclusion (PSI) was used as a threshold to define significant changes in either specific splicing events or LSVs.

### GSEA

The R package fgsea 1.18.0 (Korotkevich et al., 2021 preprint; https://bioconductor.org/packages/release/bioc/html/fgsea.html) with *n*=10^7^ gene-level permutations was used to perform the pre-ranked modification of GSEA (Subramanian et al., 2005). The pathways used were REACTOME pathways from MSigDB (GSEA, https://www.gsea-msigdb.org/gsea/). Genes were ranked by log_2_(fold change) values for each comparison of both the RNAseq (gene-level) data and the total proteomic (protein-level) data.

### Proteomics and phosphoproteomics

#### Lysis and digestion

Cells were lysed in 700 μl SDS buffer (0.1 M Tris/HCl pH 7.6, 4% SDS, 0.1 mM dithiothreitol), heated for 5 min at 95°C and sonicated (BioruptorPlus Diagenode, 30 s on/30 s off cycle for ten cycles). After centrifugation for 10 min at 10,000 ***g*** at 15°C, the proteins in the supernatant were precipitated with 4× (volumes) ice-cold acetone (−20°C) and stored at −20°C overnight. The precipitates were centrifuged for 15 min at 10,000 ***g***, the acetone was decanted, and the resulting pellets were dried for 10 min at RT. The pellet was resuspended in 500 μl denaturing buffer (8 M urea and 10 mM Tris, pH 8.5). The protein concentration was determined with a Bradford assay (Bio-Rad). The proteins were reduced with dithiothreitol (5 mM final concentration) for 60 min at 37°C and alkylated with iodoacetamide (8 mM final concentration) for 45 min at RT in the dark. Lys-C protease (Wako Chemicals, Richmond, VA, USA) was added at a ratio of 1:100 to the total protein amount and incubated at 37°C for 2 h. Urea was diluted to 2 M with 50 mM Tris pH 8.5, and the samples were incubated with trypsin (1:50 w/w) (Pierce Biotechnology, USA) overnight at 37°C at 600 rpm in a ThermoMixer (Eppendorf).

#### Desalting and TMT labeling

The peptide mixtures were acidified with formic acid (FA) to 1% final concentration (pH<3) and centrifuged at 4000 ***g*** for 5 min. The supernatants were transferred to reversed-phase C18 Sep-Pak cartridges (Waters, Milford, MA, USA) for desalting and concentration. Sep-Pak cartridges were prepared by sequential washing with methanol, 100% acetonitrile (ACN), 50% ACN/0.1% FA and 0.1% trifluoric acid (TFA) prior to loading of the peptide mixtures. Gravity was used for washing and loading of the samples. After sample loading, the Sep-Pak columns were washed with 0.1% TFA followed by 1% FA. Peptides were eluted from Sep-Pak with 50% ACN/0.1% FA in two sequential steps. The eluted peptides were dried in a Speed-Vac centrifuge (Savant SPD131DDA, Thermo Fisher Scientific), reconstituted in 50 mM HEPES, and sonicated in a water bath for 10 min. The peptide concentration was determined with a Pierce peptide assay (PI23275). Approximately 25 μg of each sample was transferred to a fresh tube for TMTpro 16plex labeling (Thermo Fisher Scientific). The TMT kit was warmed to RT, reconstituted in 41 μl of ACN, vortexed and incubated for 5 min. The peptides were labeled according to the manufacturer's instructions. To quench the reaction, 32 μl of 5% hydroxylamine was added and incubated for 15 min at RT at 1000 rpm in a ThermoMixer (Eppendorf). The labeled samples were combined and dried out in a Speed-Vac centrifuge.

#### Off-line basic reversed-phase (high pH) high-pressure liquid chromatography fractionation

The samples were reconstituted in buffer A [4.5 mM ammonium formate (pH 10) in 2% (v/v) ACN] and sonicated in a water bath for 10 min. About 90% of the TMT-labeled peptide mixture was fractionated using an Agilent 3.5-μm, 4.6×250 mm Zorbax 300 Extend-C18 column on an Agilent 1200 HPLC at 1 ml/min flow. Buffer A consisted of 4.5 mM ammonium formate (pH 10) in 2% (v/v) ACN and buffer B consisted of 4.5 mM ammonium formate (pH 10) in 90% (v/v) ACN; both buffers were adjusted to pH 10 with ammonium hydroxide. The gradient was programmed as following: 0-7 min, 0% buffer B; 7-13 min, 16% buffer B; 13-73 min, 40% buffer B; 73-77 min, 44% buffer B; 77-82 min, 60% buffer B; 82-96 min, 60% buffer B. Fractions were collected in 1 min intervals in a 96-deep-well plate. The fractions were pooled into 12 fractions (pooling all fractions that were 12 wells apart) with the pooled flow through, and dried in a Speed-Vac centrifuge overnight.

### Phosphopeptide enrichment

Phosphopeptides from each pooled fraction were enriched on IMAC beads (Ni-NTA Superflow Agarose; QIAGEN). For a 13-fraction IMAC enrichment, 160 μl of beads (320 μl of slurry) were washed three times in 1 ml water and pelleted by centrifugation at 1000 ***g*** at RT for 1 min. The beads were resuspended in 1200 μl of 100 mM EDTA for 30 min at RT with end-over-end turning, washed three times in 1 ml water, and centrifuged at 1000 ***g*** at RT for 1 min. The beads were activated with 1200 μl of 10 mM iron (III) chloride aqueous solution [solid iron (III) chloride in HPLC water] for 30 min at RT with end-over-end turning. The beads were then washed three times in 1 ml water, pelleted at 1000 ***g*** at RT for 1 min, and resuspended in 460 μl of 1:1:1 (v/v/v) ratio of ACN/methanol/0.01% (v/v) acetic acid. For each pooled fraction, 40 μl of bead slurry (10 μl of beads) was added to each of the 13 tubes. Each pooled fraction was resuspended in 190 μl of 50% (v/v) ACN/0.1% (v/v) TFA with vortexing. Once all the peptides were in solution, 285 μl of 100% (v/v) ACN/0.1% (v/v) TFA was added to the aliquoted beads and incubated for 30 min at RT at 1000 rpm. After incubation, the samples were spun down for 1 min at 1000 ***g*** at RT and the supernatant was removed. Then, 200 μl of 80% (v/v) ACN/0.1% (v/v) TFA was added to the beads.

### Elution and desalting of phosphopeptides

Peptides were desalted in 2-plug C18 (Empore C18 extraction disks) stage tips sequentially conditioned with 100% ACN, 50% (v/v) ACN/0.1% (v/v) FA and 1% (v/v) FA by centrifugation at 3000 ***g*** for 3 min. The sample on the beads was loaded onto the stage tip, centrifuged at 3000 ***g*** for 3 min at RT and washed sequentially with 80% (v/v) ACN/0.1% (v/v) TFA, 1% (v/v) FA, 500 mM K_2_PO_4_ and 1% (v/v) FA. The phosphopeptides were eluted with 50% (v/v) ACN in 0.1% (v/v) FA dried by vacuum centrifugation.

### MS analyses

The 13 fractions were reconstituted in 0.1% FA, 3% ACN in HPLC-grade water, sonicated in a water bath sonicator for 10 min and loaded on a 50 cm column (Thermo Fisher Scientific, ES903). The peptides were separated by reversed-phase chromatography using a gradient from 5% to 30% buffer B over 2 h (Buffer A: 0.1% FA in HPLC-grade water; Buffer B: 80% ACN, 0.1% FA) with a flow rate of 0.25 μl/min using an EASY-nLC 1200 system (Thermo Fisher Scientific). MS data were acquired on a Q Exactive HF mass spectrometer (Thermo Fisher Scientific) using a data-dependent acquisition top ten method, AGC target 5e5, maximum injection time of 50 ms, scan range of 350-1800 m/z and a resolution of 60,000. Tandem MS (MS/MS) was performed at a resolution of 60,000, AGC target 1e5, maximum injection time of 105 ms and isolation window 0.7 m/z. Dynamic exclusion was set to 20 s.

Raw MS data were processed using Proteome Discoverer 2.4 (Thermo Fisher Scientific) with the SEQUEST search engine (Eng et al., 1994). The MS/MS spectra were searched against the *Homo sapiens* UniProt sequence database without spliced isoforms. All MS/MS spectra were searched with the following parameters for peptide identification: acetylation (protein N-terminus) and methionine oxidation were selected as variable modifications and cysteine carbamidomethylation was selected as fixed modification. For analyses of phosphopeptides, phosphorylation (Ser, Thr, Tyr) was added as a variable modification. A maximum of two missed cleavages was allowed. Peptide spectrum matches, proteins and sites were automatically filtered to a 1% FDR by Percolator (Käll et al., 2007). Modified peptides required a minimum peptide length of at least seven amino acids.

### Quantification

TMT reporter ion intensity values were quantified from MS/MS scans using an integration tolerance of 20 ppm (Orbitrap) with the most confident centroid setting (Proteome Discoverer 2.4, Thermo Fisher Scientific) for matching peptides. For proteomic and phosphoproteomic analysis, the sum of raw reporter ion for each channel was normalized to the global median, assuming equal input loading of all channels.

MSstatsTMT workflow starts from the peptide intensities reported in Proteome Discover's PSMs.txt file. When a peptide and charge combination is measured multiple times in a sample, only the maximum intensity is kept. The log_2_ peptide intensities are median normalized assuming equal input loading of all channels. Peptide intensities are summarized to protein intensities using Tukey's median polish algorithm (Huang et al., 2020). MSstatsTMT builds protein-wise linear models based on these protein summaries, which was used for global proteomics analysis. For phosphoproteomics, we developed in-house peptide-wise linear models based on peptide summaries using the same framework.

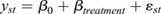


In global proteomics, *y*_*st*_ is the normalized log_2_-transformed protein intensity in sample *s* of treatment *t*, *β*_0_ is the intercept, *β*_*treatment*_ is the effect of the treatment condition *t*, and, ε_*st*_ represents the protein-wise random error terms, which are assumed to be normally distributed with mean 0 and variance *σ*^2^. In phosphoproteomics, the *y*_*st*_ is the normalized log_2_-transformed peptide intensity in sample *s* of treatment *t*, and the other notations are the same. The multiple-testing problem is corrected using the Benjamini–Hochberg FDR procedure.

Log_2_(fold change) values of phosphopeptides were subsequently normalized to the log_2_(fold change) value of their respective proteins across the same experimental comparisons to account for the effect of possible changes in abundance of the total protein level.

### Statistical analysis

Statistical tests were performed in R Studio or GraphPad Prism. The type of tests performed are defined in the figure legends or Results. Values of *n* refer to biological replicates from one experiment. Analyzed RNAseq and proteomics data are accessible in Tables S1 and S2, respectively.

## Supplementary Material

10.1242/dmm.049516_sup1 Supplementary information
