# Supplementary material for: DUX4 expression activates JNK and p38 MAP kinases in myoblasts
Source: Dis Model Mech. 2022 Oct 31;15(11):dmm049516. doi: 10.1242/dmm.049516 (PMC10655719; doi:10.1242/dmm.049516)
Supplement: Supplementary information [file dmm-15-049516-s1.pdf]

Supplemental Figure S1

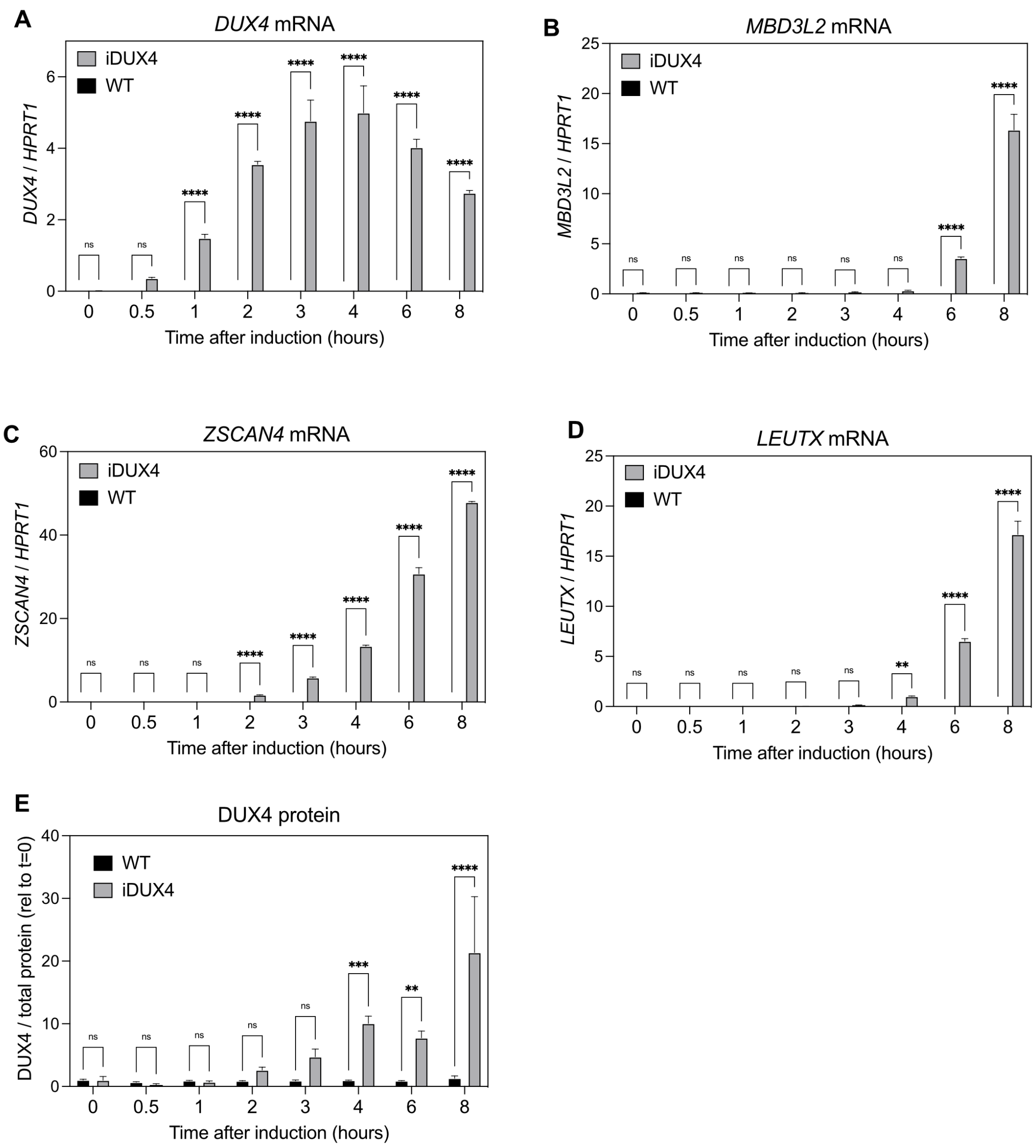

Fig. S1. Related to Figure 1. Kinetics of DUX4 induction including WT controls

(A-D) RNA was measured as in Figure 1A-D for DUX4 (A), MBD3L2 (B), ZSCAN4 (C), LEUTX (D) for both parental control MB135 myoblasts (WT, black bars) and iDUX4 MB135 myoblasts (iDUX4, gray bars).

(E) DUX4 protein was measured as in Figure 1E for both parental control MB135 myoblasts (WT, black bars) and iDUX4 MB135 myoblasts (iDUX4, gray bars). Error bars represent standard deviation. n=3, ns not significant; ** $p<0.01$; *** $p<0.001$, **** $p<0.0001$ two-way ANOVA with Tukey's multiple comparison test comparing iDUX4 and WT at each time point.

Disease Models & Mechanisms • Supplementary information

Supplemental Figure S2

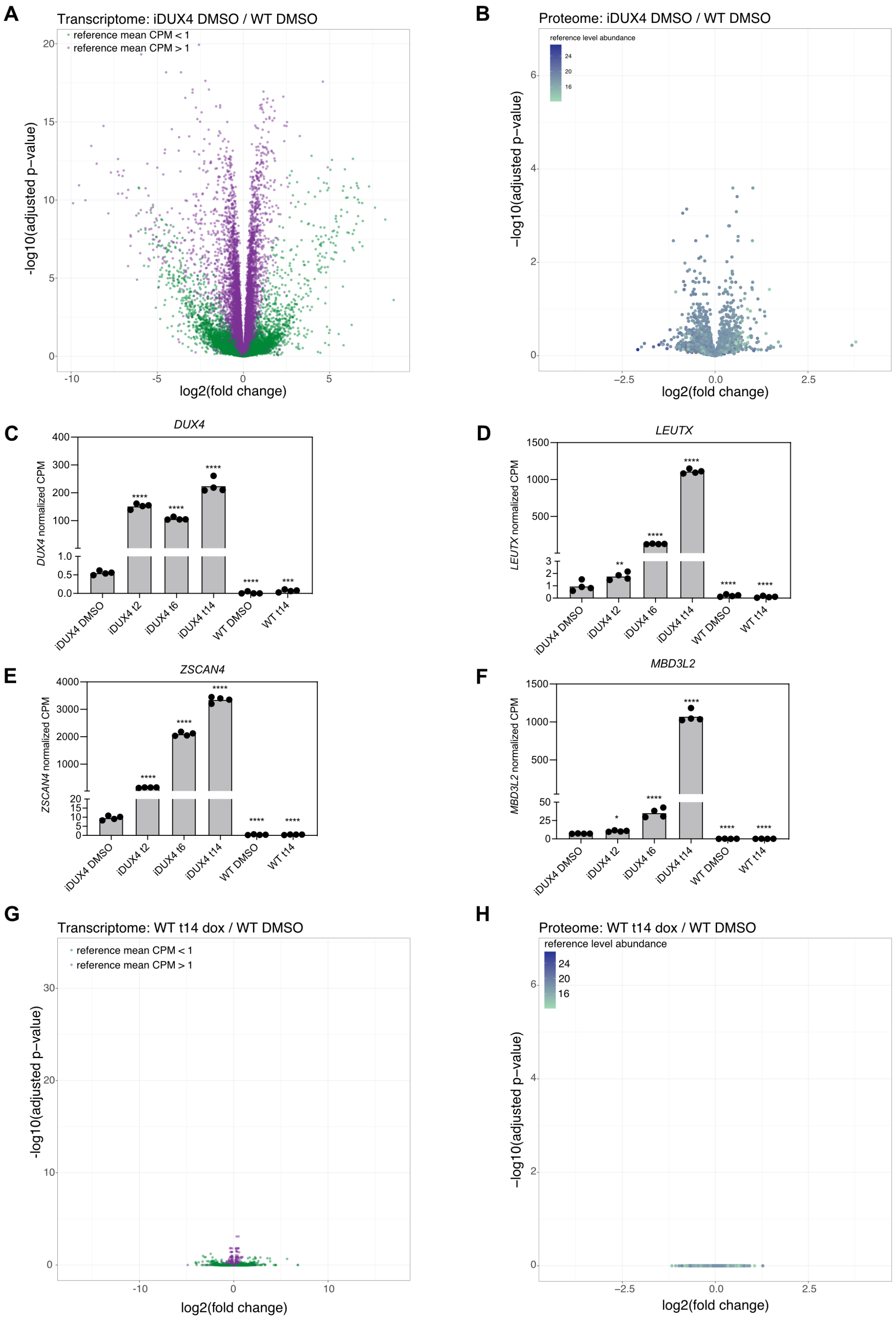

Disease Models & Mechanisms • Supplementary information

Fig. S2. Related to Figure 2. RNAseq and proteomics validation

(A) RNAseq and proteomics (B) measurements are plotted as in Figure 2 for MB135 iDUX4 (iDUX4) myoblasts treated with DMSO for 14 hours compared to MB135 parental (WT) myoblasts treated with DMSO for 14 hours.
(C-F) Normalized counts from RNAseq for *DUX4* (C) and *LEUTX* (D), *ZSCAN4* (E), *MBD3L2* 41
(F). n=4, * p<0.05, ** p<0.01, *** p<0.001, **** p<0.0001, RNAseq adjusted p-value compared to DMSO treated iDUX4 myoblasts.
(G) RNAseq and proteomics (H) measurements are plotted as in Figure 2 for MB135 parental (WT) myoblasts treated with doxycycline for 14 hours compared to MB135 parental (WT) 45
myoblasts treated with DMSO for 14 hours
Abbreviations: CPM, counter per million

Disease Models & Mechanisms • Supplementary information

Supplemental Figure S3

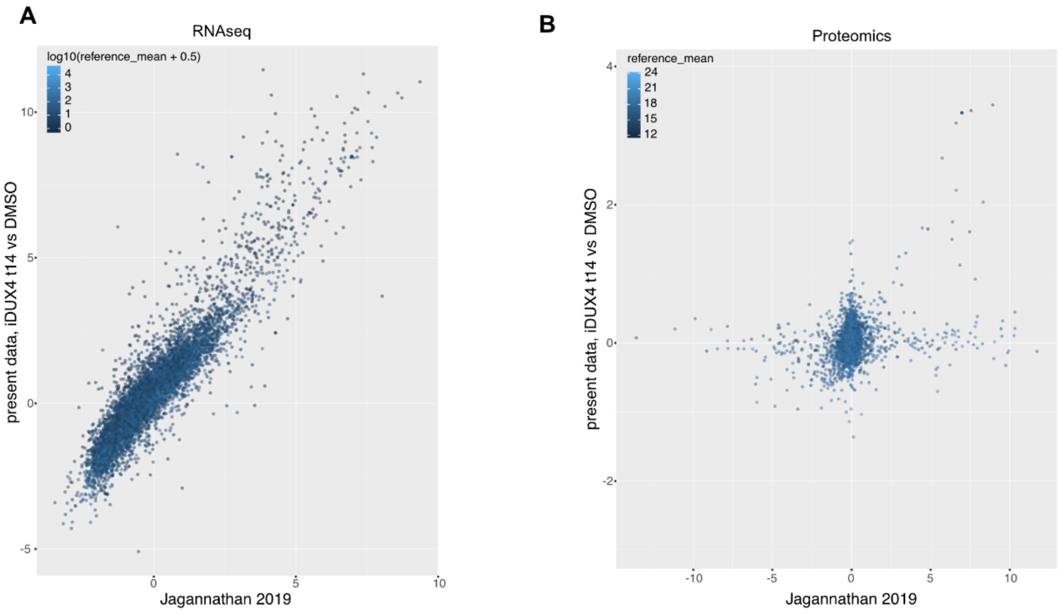

Fig. S3. Related to Figure 2. Comparison to Jagannathan et al., 2019

(A) Fold change values of mRNAs identified in both our dataset and Jagannathan et al., 2019 are plotted. Genes are colored according to their abundance in the reference samples.

(B) Fold change values of proteins identified in both our dataset and Jagannathan et al., 2019 are plotted. Proteins are colored according to their abundance in the reference samples.

Supplemental Figure S4

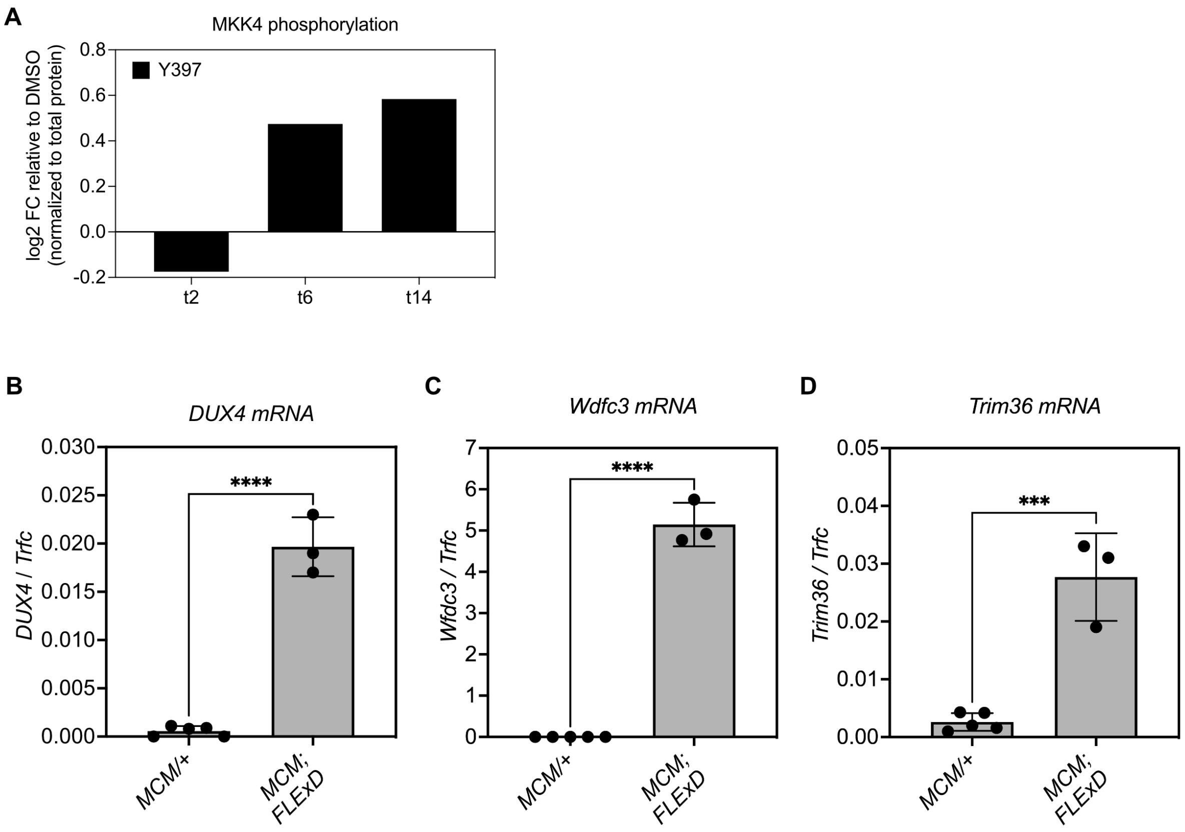

Fig. S4. Related to Figure 6. MKK4 phosphorylation and induction of DUX4 in *FLExDUX4* mice (A) Phosphorylation levels of MKK4 as measured by mass spec following induction of DUX4 relative to the uninduced controls. Measurements are normalized to total protein as in (Figure 5). (B-D) *ACTA1-MCM/+* (*MCM/+*) and *ACTA1-MCM; FLExDUX4* (*MCM; FLExDUX4*) mice were injected with 5mg/kg tamoxifen and analyzed 9 days post injection as in (Figure 6I, J). mRNA expression of *DUX4* (B), *Wdfc3* (C), and *Trim36* (D) was measured in tibialis anterior (TA) using ddPCR and normalized to *Trfc* expression. Error bars represent standard deviation. *MCM/+* n=5, *MCM;FLExD* n=3, *** p<0.001, **** p<0.0001, unpaired two-tailed T-test.

Disease Models & Mechanisms • Supplementary information

Supplemental Figure S5

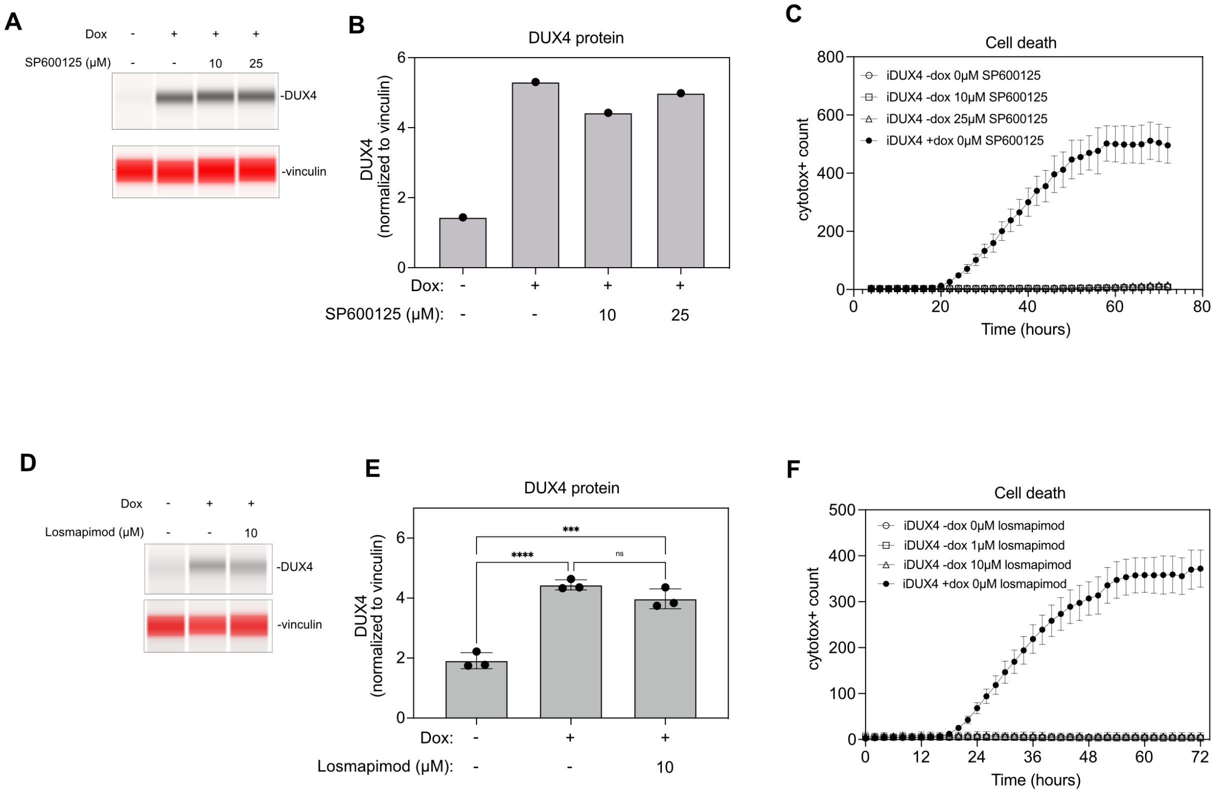

Fig. S5. Related to Figure 7. JNK/p38 inhibition do not prevent DUX4 induction in iDUX4 myoblasts

(A, B) Capillary based Western blot (A) and quantification (B) of DUX4. Vinculin was used for normalization. n=1

(C) DUX4 expression was induced in iDUX4 myoblasts and cell death was quantified as in Figure 7A. Closed circles represent cells that received doxycycline and open symbols indicate cells that did not receive doxycycline. Error bars represent 95% confidence intervals. n=8.

(D, E) Capillary based Western blot (A) and quantification (B) of DUX4. Vinculin was used for normalization. Error bars represent standard deviation. n=3, ns not significant, *** $p<0.001$; **** $p<0.0001$, one-way ANOVA with Dunnett's multiple comparisons.

(C) DUX4 expression was induced in iDUX4 myoblasts and cell death was quantified as in (C). Closed circles represent cells that received doxycycline and open symbols indicate cells that did not receive doxycycline. Error bars represent 95% confidence intervals. n=8.

Abbreviations: Dox, doxycycline

Disease Models & Mechanisms • Supplementary information

Table S1. RNAseq

Click here to download Table S1

Table S2. Proteomics and phosphoproteomics

Click here to download Table S2

Table S3. GSEA lists

Click here to download Table S3

Disease Models & Mechanisms • Supplementary information
